# Crosstalk of the Hippo/YAP pathway in the progression of oral potentially malignant disorders to oral squamous cell carcinoma: a systematic review

**DOI:** 10.3389/froh.2026.1808680

**Published:** 2026-05-25

**Authors:** Kimia Sadat Kazemi, Matheus de Castro Costa, Marina Lara de Carli, Andrew Leask, Cristiane Miranda Franca, Felipe Fornias Sperandio

**Affiliations:** 1 College of Dentistry, University of Saskatchewan, Saskatoon, SK, Canada; 2 Bauru School of Dentistry, University of São Paulo, Bauru, Brazil; 3 College of Dentistry, Federal University of Alfenas, Alfenas, Brazil; 4 Department of Biomaterial and Biomedical Sciences, School of Dentistry, Oregon Health & Science University, Portland, OR, United States

**Keywords:** biomarkers, epithelial mesenchymal transition (EMT), Hippo-YAP signalling pathway, oral potentially malignancy disorders (OPMDs), oral squamous cell carcinoma (OSCC)

## Abstract

**Objective:**

The aim of this review is to synthesize current evidence on the interaction between Hippo-YAP signaling and EMT in the malignant transformation of oral potentially malignant disorders (OPMDs) to oral squamous cell carcinoma (OSCC) and to examine their potential utility as biomarkers and therapeutic targets. Design: Following PRISMA 2020 guidelines, five electronic databases (PubMed, Web of Science, Scopus, LIVIVO, and Embase) were utilized for this search. Eligible studies included human tissue-based investigations and complementary *in vitro* experiments evaluating YAP/TAZ or EMT markers in OPMDs and OSCC. Risk of bias was assessed using QUIN, SYRCLE, and JBI tools.

**Results:**

From 2,208 records, 12 studies (26 datasets) were included. Across study designs, Hippo-YAP dysregulation and EMT activation were consistently observed across the normal to OPMD to OSCC progression. YAP nuclear localization correlated with reduced E-cadherin and increased vimentin, N-cadherin, Snail, and Slug expression. Crosstalk between YAP and MAPK/ERK, PI3K/Akt/mTOR, and Wnt/β-catenin pathways further amplified EMT signaling. High *YAP*, *hTERT*, *circEPSTI1*, and *SNAI2* expression, together with low *KLK6*, were associated with poor prognosis and increased malignant transformation risk. Pharmacologic inhibition of PI3 K/mTOR, MEK/ERK, or *LSD1* reversed EMT phenotypes in experimental models. Conclusion: Integrated activation of Hippo-YAP and EMT pathways is a pivotal event in OPMD-OSCC progression. *YAP*-centered EMT regulation shows promise as both a biomarker of malignant potential and a therapeutic target for chemoprevention and early intervention.

## 1 Introduction

Oral squamous cell carcinoma (OSCC) is the most common malignancy of the oral cavity and accounts for over 90% of oral cancers worldwide (1). Despite advances in surgery, radiotherapy, and chemotherapy, the 5-year survival rate of OSCC patients remains stagnant at approximately 50%–60% for decades (1). One of the major challenges in improving clinical outcomes arises because OSCC is often diagnosed at advanced stages when the tumor has already invaded locally and/or metastasized to cervical lymph nodes (2).

In fact, approximately 60%–70% of oral squamous cell carcinomas (OSCCs) arise from pre-existing oral potentially malignant disorders (OPMDs), which highlights the importance of accurately assessing their malignant potential (3). Evidence from longitudinal cohorts and meta-analyses indicates that mild dysplasia carries a transformation risk of approximately ≤5%, moderate dysplasia around 10%, and severe dysplasia (including carcinoma *in situ*) between 16% and 39% (4, 5). Hence, there is a pressing need to identify robust molecular biomarkers that can accurately stratify OPMDs, based on their likelihood of progression to OSCC.

One biological process that has emerged as a critical determinant of malignant transformation is epithelial-mesenchymal transition (EMT), an alteration that occurs during early carcinogenesis in essentially all premalignant conditions, including in the oral cavity (6, 7). EMT is characterized by the downregulation of epithelial markers such as E-cadherin, and the acquisition of mesenchymal markers including vimentin and N-cadherin; these alterations are concomitant with the ability of epithelial cells to become motile, invasive, and resistant to apoptosis (8).

Therefore, different signalling pathways implicated in EMT have been extensively studied in cancer. EMT is orchestrated by a set of transcription factors that repress epithelial markers and activate mesenchymal programs. Among these, the Snail family (*SNAI1, SNAI2*) and Twist (*TWIST1*) are considered master regulators of EMT. Snail proteins suppress *CDH1* (E-cadherin) transcription, therefore weakening cell-cell adhesion, while Twist plays an important role in cytoskeletal remodeling and mesenchymal gene expression; these factors drive phenotypic plasticity and interact with signaling pathways such as Hippo-YAP, Wnt/β-catenin, and PI3 K/Akt, amplifying EMT during tumor progression (8).

The Hippo-YAP signaling pathway, for instance, has gained attention as a key regulator of EMT in tumorigenesis of a wide variety of cancers, such as breast cancer, hepatocellular carcinoma, colorectal cancer, lung cancer, and head and neck squamous cell carcinoma (9–14). Hippo signaling pathway is a fundamental regulator of tissue homeostasis, organ size, and cell fate (9, 15). Its core effectors, Yes-associated protein (YAP) and transcriptional co-activator with PDZ-binding motif (TAZ), act as transcriptional coactivators that shuttle between the cytoplasm and nucleus depending on pathway activity (9). YAP functions as a central mechanotransducer: it senses extracellular matrix stiffness and cell shape via Rho/actomyosin tension, then translocates into the nucleus to regulate transcriptional programs that promote EMT, proliferation, and survival (16). In normal, unstimulated tissue, the Hippo kinase cascade (MST1/2-LATS1/2) phosphorylates transcriptional cofactors YAP/TAZ, leading to their cytoplasmic retention and subsequent proteasomal degradation (15, 17, 18). When Hippo signaling is deactivated, for example, in response to stimulation of the extracellular matrix (ECM)-stimulated mechano-transduction pathway (9), YAP/TAZ are no longer phosphorylated by MST1/2-LATS1/2 and therefore can be translocated into the nucleus to interact with TEAD transcription factors and induce the expression of genes promoting not only EMT but also proliferation and survival (15, 19).

Examples of direct YAP targets pertinent to EMT include the mesenchymal markers vimentin, N-cadherin and CCN2/CCN1. The ability of YAP to upregulate these proteins contributes to tumor invasion and metastasis in many cancers (14, 20, 21). In oral carcinogenesis, increasing evidence suggests that both Hippo-YAP dysregulation and EMT activation contribute to tumor initiation and progression (22, 23). However, despite recognition of their individual roles, the integrated contribution of these pathways in the transition from oral potentially malignant disorders (OPMDs) to oral squamous cell carcinoma (OSCC) remains poorly understood (14, 24).

Although both EMT and Hippo-YAP dysregulation are implicated in OSCC progression, their combined role in the critical transition from OPMDs to invasive carcinoma has not been systematically synthesized in the context of a review article. Furthermore, the potential of Hippo-YAP and EMT markers as predictors of malignant transformation in high-risk OPMDs, as well as their therapeutic targeting for chemoprevention, remains underexplored. The objectives of this focused systematic review are to: summarize existing evidence on EMT activation and Hippo-YAP dysregulation in OPMDs and OSCC, evaluate their functional interplay in driving malignant progression, and assess their utility as prognostic biomarkers and potential therapeutic targets. By clarifying these mechanisms, this review seeks to advance understanding of early oral carcinogenesis and to highlight opportunities for improving early detection, risk stratification, and intervention strategies.

## 2 Materials and methods

### 2.1 Research question and protocol

This systematic review was conducted to address the research question:

“What is the role of the Hippo-YAP signaling pathway or epithelial-mesenchymal transition (EMT) in the development and progression of oral potentially malignant disorders (OPMDs) to oral squamous cell carcinoma (OSCC)?” The review protocol was developed according to the PRISMA 2020 guidelines for systematic reviews (25).

### 2.2 Eligibility criteria

- Inclusion criteria

The eligibility criteria were defined using the PICOS framework:

Population (P): Patients or human tissue samples with OPMD such as leukoplakia, oral epithelial dysplasia (OED), oral submucous fibrosis (OSF), oral lichen planus or OSCC.

Intervention/Exposure (I): Activation, inhibition, or dysregulation of the Hippo-YAP signaling pathway and/or EMT.

Comparator (C): Normal oral mucosa or OPMDs without progression, when available.

Outcomes (O): Evidence of malignant progression (histopathological severity, OSCC development), altered expression of Hippo-YAP or EMT markers, cellular behaviors (invasion, migration), and clinical outcomes (metastasis, recurrence, survival).

Study design (S): Human studies including cross-sectional, retrospective, and prospective analyses of tissue samples, as well as *in vitro* experiments using human-derived cell lines. *In vivo* animal studies were excluded unless they included clearly separable *in vitro* human data.

ii. *Exclusion criteria*

(i) non-human studies, (ii) non-English language publications, (iii) lack of access to full text, and (iv) editorials, commentaries, and case reports not relevant to the research question.

iii. Information sources and search strategy

Electronic searches were conducted in PubMed, Web of Science, Scopus, LIVIVO, and Embase up to July 6, 2025. Search strategies combined controlled vocabulary (MeSH/Emtree terms) and free-text keywords related to YAP, Hippo signaling pathway, EMT, OPMDs, and OSCC (Table 1**)**.

iv. Study selection

**Table 1:** Search strategies applied across five electronic databases (pubMed, Web of science, scopus, livivo, and embase) to identify studies investigating the hippo-YAP signaling pathway and epithelial-mesenchymal transition (EMT) in oral squamous cell carcinoma (OSCC) and oral potentially malignant disorders (OPMDs).

Database	Search strategy	Results	Date
PubMed	(“YAP"[ti] OR “YAP1"[ti] OR “Hippo Signaling Pathway"[ti] OR “EMT"[ti] OR “epithelial-mesenchymal transition"[ti]) AND (“oral squamous cell carcinoma"[tiab] OR “oral leukoplakia"[tiab] OR “oral epithelial dysplasia"[tiab] OR “oral potentially malignant disorders"[tiab] OR “oral precancer"[tiab] OR “OPMD"[tiab] OR “OSCC"[tiab])	286 results	Nov 6, 2025
Web of science	TI=(“YAP” OR “YAP1” OR “Hippo Signaling Pathway” OR “EMT” OR “epithelial-mesenchymal transition”) AND TS=(“oral squamous cell carcinoma” OR “oral leukoplakia” OR “oral epithelial dysplasia” OR “oral potentially malignant disorders” OR “oral precancer” OR “OPMD” OR “OSCC”)	295	Nov 6, 2025
Scopus	TITLE(“YAP” OR “YAP1” OR “Hippo Signaling Pathway” OR “EMT” OR “epithelial-mesenchymal transition”) AND (TITLE-ABS(“oral squamous cell carcinoma” OR “oral leukoplakia” OR “oral epithelial dysplasia” OR “oral potentially malignant disorders” OR “oral precancer” OR “OPMD” OR “OSCC”))	293	Nov 6, 2025
Livivo	(“YAP” OR “YAP1” OR “Hippo Signaling Pathway” OR “EMT” OR “epithelial-mesenchymal transition”) AND (“oral squamous cell carcinoma” OR “oral leukoplakia” OR “oral epithelial dysplasia” OR “oral potentially malignant disorders” OR “oral precancer” OR “OPMD” OR “OSCC”)	1020 results 996 English 19 Chinese 1 Russian	Nov 6, 2025
Embase	(“YAP” OR “YAP1” OR “Hippo Signaling Pathway” OR “EMT” OR “epithelial-mesenchymal transition”).ti. AND (“oral squamous cell carcinoma” OR “oral leukoplakia” OR “oral epithelial dysplasia” OR “oral potentially malignant disorders” OR “oral precancer” OR “OPMD” OR “OSCC”).ti,ab.	314	Nov 6, 2025

Searches used controlled vocabulary and keyword combinations tailored to each database.

**Figure 1:**
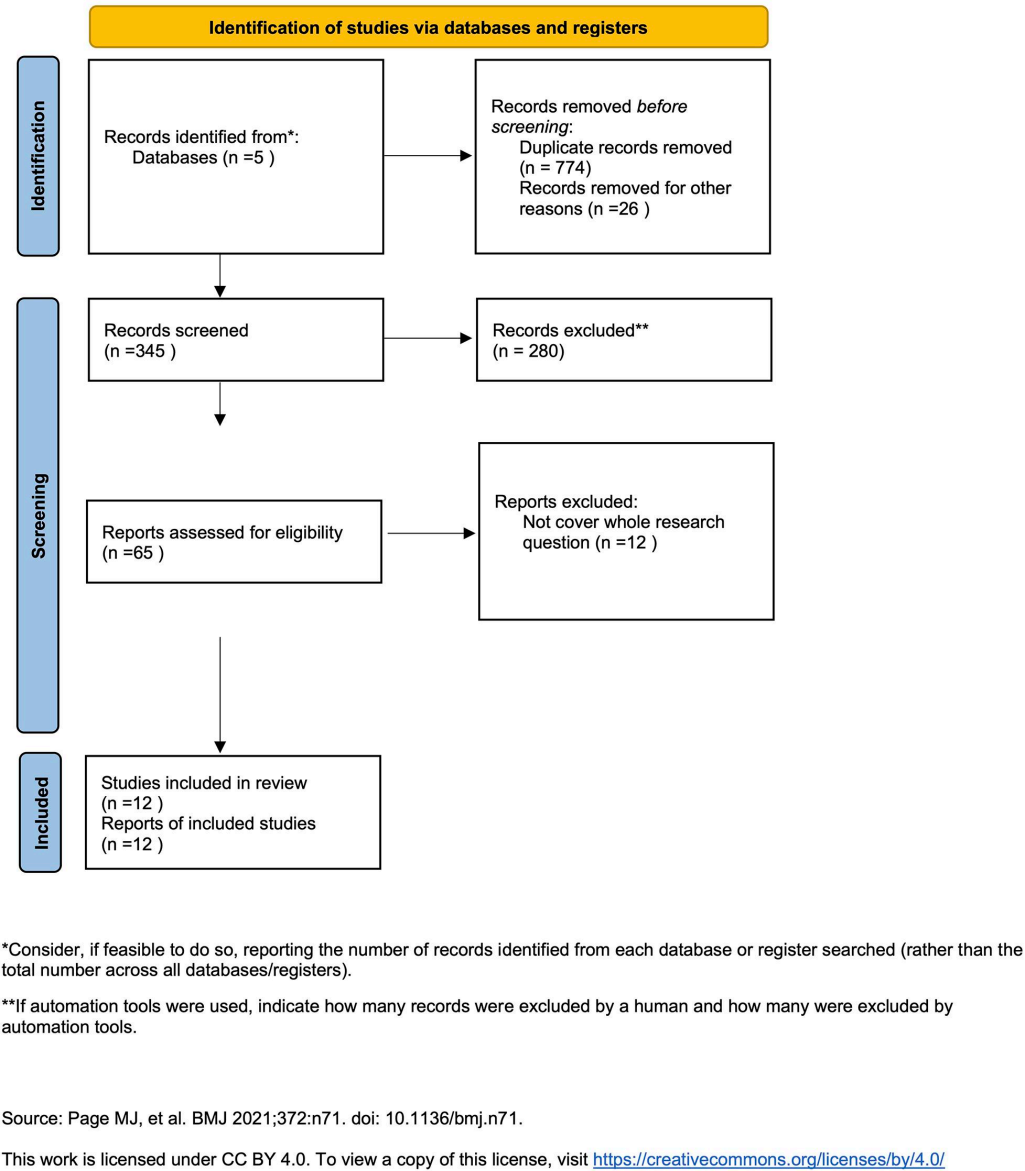
Criteria for the selection of articles. PRISMA 2020 flow diagram for new systematic reviews which included searches of databases and registers only.

All records were imported into a reference manager, and duplicates were removed. Titles and abstracts were screened independently by two reviewers, against eligibility criteria. Full texts of potentially relevant studies were then retrieved and assessed for inclusion. Disagreements were resolved by discussion, and inter-rater reliability was calculated with Cohen's kappa (*κ* = 0.80), indicating substantial agreement (26). A PRISMA 2020 flow diagram was used to document the screening and selection process (Figure 1).

### Data extraction

Data were extracted independently by two reviewers using a standardized extraction form. Extracted items included: study ID, authors, year, country, study design, population/sample size, OPMD or OSCC subtype, Hippo-YAP or EMT markers studied, method of assessment such asimmunohistochemistry (IHC), quantitative-PCR (qPCR) or Western blot (WB), intervention or exposure, comparator, outcomes (progression, invasion, prognosis), and key findings. For *in vitro* studies, details of cell lines, genetic manipulations, and pharmacological treatments were also recorded.

iv. Risk of bias Assessment

Risk of bias was assessed independently by two reviewers according to study type:

*In vitro* studies: QUIN tool.

*In vivo* studies: SYRCLE RoB tool.

Cross-sectional and cohort studies: JBI Critical Appraisal Checklist for Analytical Cross-Sectional Studies.

Each domain was judged as low, moderate, or high risk of bias, and disagreements were resolved by consensus. Results of the risk of bias assessment are summarized in Supplementary Tables 1–4.

## 3 Results

### 3.1 Systematic literature search

The initial database search retrieved a total of 2208 records (PubMed = 286, Web of Science = 295, Scopus = 293, LIVIVO = 1020, and Embase = 314). After the removal of duplicates, 345 unique articles remained. Screening of titles and abstracts excluded 280 studies that did not meet the eligibility criteria, leaving 65 articles for full-text review. Following detailed evaluation, 12 studies met all inclusion criteria and were included in the final qualitative synthesis (Figure 1). The inter-reviewer agreement for the screening process was substantial (Cohen's *κ* = 0.80), confirming high consistency among reviewers.

### 3.2 Study characteristics

A total of 12 eligible articles were included in this systematic review. However, several publications contained more than one distinct experimental or analytical component; therefore, each dataset or sub-study was analyzed separately, resulting in a total of 26 studies for synthesis.

**Figure 2:**
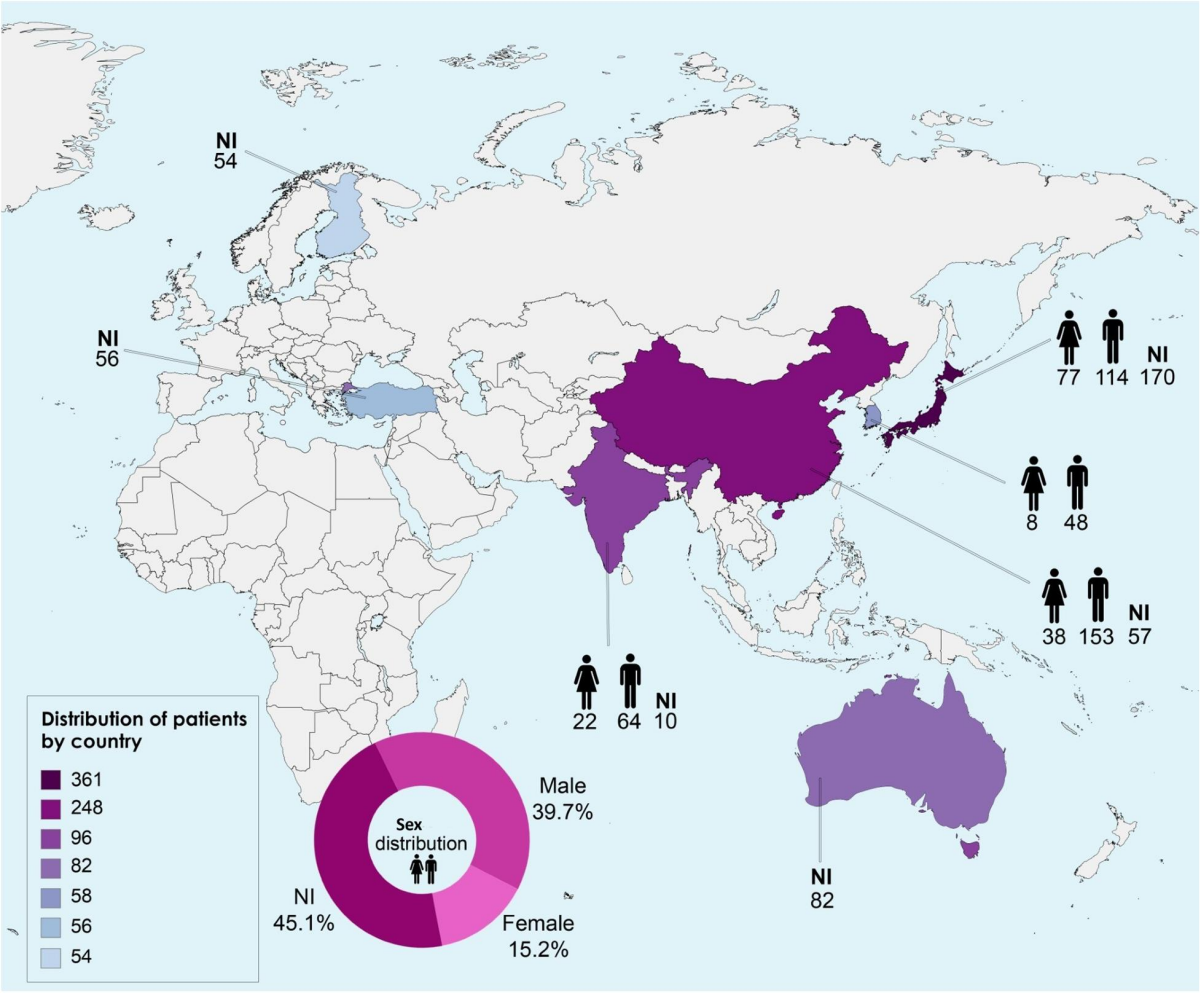
Demographic data of included patients and geographical location of the included studies.

Among these, 3 were *in vivo*, 8 were *in vitro*, 1 was a cohort study, and 14 were cross-sectional human tissue-based analyses (Table 2). Most investigations focused on YAP1 and TAZ as the core effectors of the Hippo signaling pathway and examined their association with canonical EMT markers, including E-cadherin, N-cadherin, vimentin, Snail, and Slug. The included studies were published between 2010 and 2025, with the majority originating from Asia (China, India, and Japan) and a smaller number from Europe and North America (Figure 2). The principal analytical methods used to evaluate YAP/EMT activation were IHC, qPCR, and WB.

**Table 2:** Data extraction summary for all eligible studies, detailing study methodology, patient and tissue characteristics, molecular targets evaluated, and main outcomes associated with Hippo-YAP signaling and epithelial-mesenchymal transition.

Cohort studies															
Author, Year	Study design	Setting	Population	Inclusion criteria	Exclusion criteria	Exposure/Biomarker	Comparison group	Follow-up duration	Outcome(s)	Statistical analysis	Effect size	Main findings	Confounders adjusted	Limitations	Interpretation
Shang et al., (2023) (27)	Retrospective cohort analysis (clinical follow-up dataset, GSE26549)	Clinical cohort from GEO (Saintigny et al., 2011) with 5-year follow-up	Patients with OLK; number not explicitly restated in this article, but GSE26549 includes 86 OLK patients with follow-up	OLK patients with clinical and molecular data, followed for malignant transformation	Healthy controls excluded (from GSE85195 dataset)	*SNAI2* expression (high vs. low, divided by mean expression)	High *SNAI2* expression vs. low *SNAI2* expression	5 years (time to malignant transformation of OLK to OSCC)	Primary outcome: malignant transformation of OLK to OSCC	Kaplan–Meier survival curves, log-rank test; multivariable Cox proportional hazards regression (adjusted for confounders); ROC curve; nomogram	High *SNAI2* expression increased risk of OLK malignant transformation: HR = 2.50 (95% CI: 1.08–5.79*), p =* 0.033	Patients with high *SNAI2* expression had significantly higher malignant transformation risk compared to low *SNAI2* (*p =* 0.012, KM analysis). *SNAI2* expression was the strongest predictor in the Cox model and improved model AUC.	Age, sex, and other clinical factors	Cohort data derived from a single dataset (GSE26549), relatively small number of malignant transformations, possible selection bias.	High *SNAI2* is an independent risk factor for OLK malignant transformation and may serve as a predictive biomarker for early detection and intervention

Cross sectional studies											
Citation	Setting	Population/Sample Source	Clinical Data	Exposure/Risk Factors Considered	Comparators	Techniques/Methods	Outcomes Measured	Key Findings	Interpretation	Funding	Limitations (noted by authors)
Chatterjee et al., Eur J Cell Biol, (2021) (28)	Guru Nanak Institute of Dental Sciences and Research (GNIDSR), Kolkata, India	Human biopsy tissues: Normal oral mucosa (n = 10, from 3rd molar extraction flaps, no oral habits) OSF (n = 12, areca nut chewers) OSCC (*n* = 14, many with tobacco/areca habits)	OSF patients: Age 20–40 yrs, 66% male; all areca nut chewers. OSCC patients: Age 30–50 yrs, 55% female; habits included tobacco (33%), areca nut (41%), smoking (25%), mixed habits (16%).	Areca nut chewing, tobacco, smoking (collected as habits in)	Normal mucosa vs. OSF vs. OSCC	Histopathology (H&E, Mallory's trichrome) Immunohistochemistry (HIF-1α, 4-HNE, E-cadherin, vimentin, β-catenin, ERK, ALDH-1, CD133, Shh, Gli-1, Survivin) FTIR spectroscopy (lipid oxidation, ROS marker) Janus Green-B staining (mitochondrial disruption) Scanning electron microscopy (SEM) Atomic force microscopy (AFM)	Oxidative stress: ↑ lipid peroxidation (4-HNE), ↑ HIF-1*α* EMT markers: ↓ E-cadherin, ↑ vimentin, ↑ β-catenin (cytoplasmic & nuclear diffusion) Fibrosis/Matrix changes: Dense collagen in OSF, degraded collagen in OSCC (MT staining, AFM) Stemness markers: ALDH-1, CD133 positivity in OSCC metastatic islands (absent in normal/OSF) Pathway: Up-regulation of Shh/Gli-1 axis, correlation with Survivin	Hypoxia + oxidative stress strongly associated with OSF & OSCC (*r* = 0.98 between HIF-1*α* and 4-HNE). Type II EMT (fibrosis) in OSF; Type III EMT (metastasis** +** stemness) in OSCC. Shh/Gli-1 signaling upregulated, linked with Survivin expression (*r* = 0.93).	Cross-sectional evidence that hypoxia-induced oxidative stress drives EMT, fibrosis, stemness, and OSF→OSCC progression via Shh/Gli-1 axis. Authors propose it as a therapeutic target	DBT-RA Program in Biotechnology & Life Sciences; Dept. of Science and Technology, Govt. of West Bengal, India	Small sample size (12 OSF, 14 OSCC), cross-sectional design (no longitudinal transformation follow-up).
Zhao T et al. International Journal of Oncology (2015) (18)	Hospital of Stomatology, Sun Yat-sen University, Guangzhou, China	37 OSCC samples, 15 OED, and 10 NOM.	Age, gender, tumor location, TNM classification, histological grade (32 well-differentiated, 5 moderately differentiated OSCC).	hTERT expression levels (cytoplasmic and nuclear) measured by IHC; correlation with clinicopathological features (tumor size, grade, nodal status).	NOM OED OSCC|	IHC for hTERT hTERT expression (cytoplasmic & nuclear LS) Association with clinicopathological variables (age, sex, location, T status, N status, histology). SI, LI, LS (LS = LI × SI)	hTERT expression (cytoplasmic & nuclear LS) Association with clinicopathological variables (age, sex, location, T status, N status, histology)	Cytoplasmic hTERT LS significantly increased from NOM (53.8) → OED (141.4) → OSCC (187.6) (*p*=0.0006Nuclear hTERT LS increased from NOM (123.8) → OED (217.6) → OSCC (215.0), with no significant difference between OED and OSCC. Higher nuclear hTERT associated with larger tumors (T3/T4 vs. T1/T2, *p* = 0.005) Cytoplasmic hTERT significantly higher in moderately vs. well-differentiated OSCC (*p* = 0.003). No correlation with age, gender, location, or nodal status	hTERT expression progressively increases from NOM → OED → OSCC. High hTERT correlates with aggressive clinicopathological features, suggesting its role as a biomarker for early malignant transformation and poor prognosis.	hTERT is upregulated along the transformation axis: staining intensity/labeling scores rose from normal oral mucosa (NOM) → oral epithelial dysplasia (OED) → OSCC (strongest change in the cytoplasmic compartment). Clinicopathologic correlations: Higher cytoplasmic hTERT in moderately vs. well-differentiated OSCC (worse differentiation ↔ more hTERT). Higher nuclear hTERT in larger tumors (T3-T4) vs. smaller (T1-T2). No significant association with age, sex, site, or nodal status. Implication: hTERT distribution/intensity may serve as a biomarker of malignant transformation and aggressiveness in the oral epithelium	Did not fully stratify patients by demographics or risk habits. Tissue study only shows association, not causation. Small number of moderately differentiated OSCC samples. Functional mechanisms clarified only *in vitro* (not in this cross-sectional tissue study)
Gupta et al., *Journal of Cancer Research and Therapeutics* (2022, published (2023) (29)	Department of Oral and Maxillofacial Pathology and Microbiology, I.T.S. Center for Dental Studies and Research, Ghaziabad, India	10 histopathologically confirmed cases of OSMF 5 cases of OSMF transforming into OSCC Archival paraffin-embedded tissue samples	Mean age: 34.8 ± 2.1 years Majority male patients Habits: predominantly tobacco smoking + chewing (53.5%), mostly <10 years duration	Areca nut chewing (primary etiological agent of OSMF) Hypoxia-mediated signaling pathways (via HIFs) affecting EMT & angiogenesis	Group 1 = OSMF cases Group 2 = OSCC arising from OSMF	IHC for α-SMA, Vimentin, E-cadherin, Factor VIII Semi-quantitative, qualitative, and quantitative immunoscoring Statistical tests: ANOVA, Pearson's Chi-square, Mann–Whitney	α-SMA positive myofibroblasts (superficial vs. deep stroma, intensity, topography) Vimentin LI and SI E-cadherin expression (extent, intensity, localization) Factor VIII mean vessel density (angiogenesis marker) Correlations between markers (Pearson & Spearman)	α-SMA: Myofibroblasts increased overall from OSMF to OSCC; superficial stroma higher in OSMF, deep stroma higher in OSCC. Vimentin: LI increased from OSMF (18.0) → OSCC (22.2). Stronger staining seen in OSCC. E-cadherin: Reduced in OSCC; membranous localization in OSMF (80%), cytoplasmic in OSCC (40%). Negative correlation with Vimenti Factor VIII: Mean vessel density increased from OSMF (13.74) → OSCC (17.03). α-SMA deep stroma positively correlated with Factor VIII (0.495); α-SMA superficial stroma positively correlated with Vimentin (0.508); E-cadherin intensity negatively correlated with Vimentin intensity (−0.538)	EMT and angiogenesis play a central role in malignant transformation of OSMF. Loss of E-cadherin and increased α-SMA, Vimentin, and Factor VIII suggest a hypoxia-driven EMT/angiogenesis axis that promotes OSCC development.	α-SMA (deep stroma) positively correlates with MVD (r≈0.50): more myofibroblasts in deep tissue ↔ more vessels. α-SMA (superficial stroma) positively correlates with Vimentin (r≈0.51): stromal activation tracks with epithelial mesenchymal shift. E-cadherin intensity negatively correlates with Vimentin intensity (Spearman =−0.54): classic EMT signature. E-cadherin (semi-quantitative) negatively correlates with α-SMA (r≈ −0.16, small but in EMT-consistent direction)	Small sample size (10 OSMF, 5 OSCC). Archival tissue-based (retrospective, limited clinical data). Factor VIII does not distinguish hypoxia-driven vs. ischemia-driven angiogenesis. Findings are associative; mechanistic insights require molecular validation.
Gupta et al., Head and Neck Pathology (2025) (30)	Department of Oral Pathology and Microbiology, Post Graduate Institute of Dental Sciences (PGIDS), Rohtak, Haryana, India	70 FFPE tissue sections: 10 normal oral mucosa (NOM), 30 oral epithelial dysplasia (OED), 30 OSCC. Samples from archival blocks (2020–2022) + new incisional biopsies (2022–2023).	Mean age: NOM = 26.5 yrs; OED = 49.5 yrs; OSCC = 50.7 yrs. Male predominance: NOM 6/10, OED 26/30, OSCC 25/30. Common sites: buccal mucosa (OED 50%, OSCC 33.3%).	OSCC linked to OPMDs (leukoplakia, erythroplakia) Hippo/YAP signaling dysregulation hypothesized in carcinogenesis PARK2 as tumor suppressor, possible regulator of Hippo/YAP pathway.	NOM vs. OED vs. OSCC. Within OSCC: well-, moderately-, and poorly-differentiated. Within OED: mild, moderate, severe dysplasia.	IHC for YAP (nuclear/cytoplasmic) and PARK2 (cytoplasmic). Scoring: % positive cells (0–3) × intensity (0–3), final score 0–9 → categorized as Low (<6) or High (≥6). Analysis: Chi-square, Fisher's exact test, Spearman correlation, Cox regression, Kaplan–Meier survival.	Expression levels of YAP & PARK2. Correlation between markers. Association with clinico pathological parameters (tumor size, stage, grade, nodal status). Survival analysis (18-month follow-up).	YAP: Increased expression from NOM → OED → OSCC (*p=*0.025). High YAP in 56.7% OSCC cases vs. 23.3% OED and 30% NOM. Higher nuclear YAP correlated with advanced tumor size (*p* =0.034), stage (*p* =0.034), and poorer differentiation (WDSCC vs. PDSCC, *p* = 0.0198). - PARK2**:** Decreased expression from NOM → OED → OSCC (*p=*0.005). High PARK2 in 90% NOM, but only 36.7% OED and 33.3% OSCC. - Correlation: Moderate inverse correlation between PARK2 and YAP in OSCC (*r* = -0.381, *p* = 0.038). - **Survival:** Kaplan–Meier showed trend of worse survival with high YAP/low PARK2, but not statistically significant (YAP *p* = 0.65; PARK2 *p* = 0.87). Median OSCC survival ∼9 months	YAP overexpression and PARK2 depletion progress stepwise from NOM to OED to OSCC. Concomitant IHC expression could serve as diagnostic & prognostic biomarkers. Suggests PARK2 modulates Hippo/YAP axis, promoting YAP degradation and apoptosis; its loss permits carcinogenesis	YAP: Higher expression with larger tumor size (T3-T4, *p* = 0.034). Higher in advanced clinical stage (III-IV, *p* = 0.034). Grading: YAP expression increased from WDSCC → MDSCC → PDSCC (*p=0.001*). PARK2:No significant associations with size, nodal status, or stage. Decline from WDSCC → PDSCC noted but not significant (*p=0.350*). Survival (Kaplan–Meier, 18-month follow-up) YAP high and PARK2 low trended to poorer survival, but results were not statistically significant (YAP *p* = *0.65*; PARK2 *p* = *0.87*)	Small sample size (*n* = 70, only 30 OSCC). Short follow-up (18 months). Single technique (IHC only, no molecular assays). Survival differences not statistically significant
Chaw et al. (2012) (31)	University of Queensland & Melbourne Dental School	100 FFPE oral biopsy specimens (18 normal, 27 mild dysplasia, 8 moderate-severe dysplasia, 47 OSCC)	Histologically confirmed diagnosis (normal, OED, OSCC) between 2000 and 2008	Not explicitly stated; implicit exclusion of poor-quality or unclassifiable samples	EMT biomarkers: E-cadherin, β-catenin, APC, Vimentin (IHC, RT-PCR, organotypic model)	Normal vs. dysplasia vs. OSCC tissues	Archival (2000–2008), no longitudinal patient follow-up (cross-sectional tissue-based)	Malignant transformation markers; EMT evidence; correlation with histopathology	Mann–Whitney test, Spearman correlation, RT-PCR quantification	Not expressed as OR/HR; reported relative IHC scores and mRNA fold changes	↓E-cadherin, ↑Vimentin with progression; β-catenin shifted from membranous to cytoplasmic/nuclear; APC expression ↓ in mild dysplasia but ↑ in OSCC; strong correlation between Vimentin and β-catenin nuclear localisation
Kaneko et al. (2017) (Oral Oncology 75:148–15) (32)	Kyushu University Hospital, Japan	111 patients with primary OSCC; OSCC cell lines (HSC-2, HSC-3, SQUU-A, SQUU-B, SQUU-BO, SQUU-BC, SAS)	Histologically confirmed primary OSCC	Histologically confirmed primary OSCC	KLK6, ΔNp63, PAR1, PAR2 expression (IHC, RT-PCR, WB)	KLK6 high vs. low expression at invasive tumor front	5 years (cause-specific survival)	KLK6 expression pattern, EMT markers, recurrence, metastasis, survival	Chi-square, Mann–Whitney U, Kaplan–Meier, Cox regression	HR = 4.01 (95% CI: 1.79–9.59), *p* = 0.0007 for low KLK6 and poor prognosis	Low KLK6 at invasive front linked with higher local recurrence, lymph node/distant metastasis, poorer survival; ΔNp63-KLK6-PAR2 promotes proliferation, while PAR1 upregulation at invasive front drives EMT/invasion
Hong et al., (2018) (Human Pathology, 80:123–129) (33)	Seoul National University Dental Hospital, Korea (1999–2005)	56 patients with surgically treated OSCC (48M, 8F; mean age 57.7y)	Histologically confirmed OSCC with available H&E and paraffin-embedded tissue	Not explicitly stated (likely excluded poor-quality tissue or incomplete records)	Tumor budding (H&E) and EMT regulators (Snail, Twist via IHC)	Tumor budding positive (≥3 buds at invasive front) vs. negative (<3 buds)	5-year survival data retrieved	Tumor budding, lymph node metastasis, overall survival, Snail/Twist expression	Chi-square/Fisher's exact test, Kaplan–Meier, log-rank test	Tumor budding present in 19/56 (33.9%). Associated with LN metastasis (*p* = 0.001) and poor OS (*p* = 0.002). Snail+ associated with LN metastasis (*p* < 0.001) and poor OS (*p* = 0.024). Twist+ associated with LN metastasis (*p* = 0.002), poor differentiation *(p* = 0.011), and poor OS (*p* = 0.025). Tumor budding correlated with Snail (*p* = 0.003) and trend with Twist (*p*=0.08)	None explicitly adjusted (no multivariate analysis beyond KM/log-rank)
Zheng et al. (2018) (Oncology Research) (34)	First Affiliated Hospital of Zhengzhou University, China	10 OSF tissues and 10 NOM	Histologically confirmed OSF and NOM; informed consent obtained	Not reported	p53 protein expression; TP53 promoter methylation	OSF vs. NOM	p53 expression (Western blot)	Very small sample size (*n* = 20), single center, no clinical outcomes	TP53 promoter hypermethylated in OSF (40%) vs. none in NOM (0%)	Very small sample size (*n* = 20), single center, no clinical outcomes	OSF shows reduced p53 expression associated with TP53 promoter hypermethylation, supporting its role in malignant transformation risk
Liu et al. (2021) (J Cell Biochem) (35)	Shanghai Ninth People's Hospital, China	Microarray cohort: NOM (*n* = 3), low-risk OL (*n* = 4), high-risk OL (*n* = 4), Stage I/II OSCC (*n* = 6). Validation cohort: NOM (*n* = 6), low-risk OL (*n* = 33), high-risk OL (*n* = 27), Stage I/II OSCC (*n* = 27). Total OL patients followed: 60 (33 low-risk, 27 high-risk)	Histologically confirmed OL or OSCC; WHO criteria; untreated prior to biopsy/surgery; written consent	Not reported	lncRNA LOLA1 (n3, 86, 251) expression; EMT markers (E-cadherin, Vimentin, N-cadherin)	NOM vs. low-risk OL vs. high-risk OL vs. OSCC	Mean follow-up 38.5 months (for OL cohort, *n* = 60)	LOLA1 expression (qRT-PCR, FISH) Association with OL malignant progression (Kaplan–Meier) In OL cohort, 5/60 (8.3%) progressed to OSCC, all in high-risk group. LOLA1 overexpression strongly associated with malignant progression: 28.6% progression in LOLA1-high vs. 2.2% in LOLA1-low (*p* = 0.001)	Microarray, qRT-PCR, FISH, survival analysis	Small sample (*n* = 60 OL, 5 progression events); single-center; relatively short follow-up; validation needed.	LOLA1 is a predictive biomarker for OL malignant transformation; high LOLA1 expression may indicate elevated risk of progression to OSCC
Wang et al. (2020) (Cell Death & Disease) (36)	Xiangya Hospital, Central South University, China	162 paired biopsies (OSCC + OSF) + 38 NBM controls; 154 OSCC + OSF patients with full clinicopathological data	OSCC with histologically confirmed background OSF; surgical treatment; informed consent	<20 or >65 years; prior chemo/radiotherapy; biological/TCM treatment; incomplete follow-up; other malignancies or systemic immune disease	CircEPSTI1, miR-942-5p, LTBP2 expression; EMT markers (E-cadherin, N-cadherin, Vimentin)	NBM vs. OSF vs. OSCC (with OSF)	Mean follow-up not specified; survival analysis reported	CircEPSTI1, miR-942-5p, LTBP2 expression (qRT-PCR, IHC, FISH)	Correlation with T/N/TNM stage	High LTBP2 → worse OS & PFS CircEPSTI1 expression increased sequentially NBM → OSF → OSCC Low miR-942-5p → worse OS & PFS High LTBP2 → worse OS & PFS	Single-center; Chinese cohort only; limited follow-up details; mechanistic links mainly inferred from *in vitro*/*in vivo* assays circEPSTI1/miR-942-5p/LTBP2 axis is a biomarker triad for OSCC in OSF, predicting progression and poor survival.
Ono et al., (2019) (Int J Med Sci) (23)	Okayama University, Japan	270 cases: 20 normal oral mucosa, 20 epithelial hyperplasia, 50 low-grade OED, 50 high-grade OED, 50 CIS, 80 OSCC	Histologically confirmed lesions; WHO criteria for dysplasia and CIS; biopsy/excision samples	Prior chemo, radiotherapy, or immunotherapy	YAP and ΔNp63 expression (IHC)	Normal → hyperplasia → low-grade OED → high-grade OED → CIS → OSCC	Median follow-up not specified; OS and DFS assessed in OSCC	YAP immunolabeling scores	% ΔNp63-positive cells	IHC (YAP, ΔNp63), scoring system (0–3), Kaplan–Meier survival analysis	YAP and ΔNp63 increased progressively from normal/hyperplasia → OED → CIS → OSCC - High-grade OED & CIS had significantly higher YAP/ΔNp63 than low-grade OED (*p* < 0.05) - In OSCC: YAP/*Δ*Np63 expression varied with histopathological differentiation; higher in poorly/moderately differentiated tumors
Chen et al. (2024) (J Clin Pathol) (37)	School & Hospital of Stomatology, Wuhan University, China	48 OSCC, 78 OPMDs (70 OLK, 5 lichenoid lesions, 3 oral erythema), 25 normal oral tissues	Histologically confirmed OPMDs and OSCC; WHO criteria; untreated prior to sampling; informed consent	Not reported	Lipid droplet (LD)markers: Caveolin-2, Perilipin-3; Lipid metabolism: FABP5; EMT marker: E-cadherin	Normal vs. OPMDs vs. OSCC; within OPMDs stratified by dysplasia grade; OSCC stratified by TNM/recurrence	Median not reported; DFS and OS explored in OSCC	LDs accumulation (Oil Red O staining) Caveolin-2, Perilipin-3, FABP5, E-cadherin (IHC, IOD quantification)	Oil Red O staining; IHC; ImageProPlus quantification; Spearman correlation; Kaplan–Meier/log-rank	LDs accumulated early in OPMDs and OSCC vs. normal (*p* < 0.05) Caveolin-2, Perilipin-3, FABP5 ↑ progressively normal → OPMDs → OSCC E-cadherin ↓ with increasing dysplasia/grade (negative correlation with caveolin-2 & FABP5)	LDs accumulate early in OPMDs; caveolin-2 and FABP5 link lipid metabolism to EMT and predict recurrence; caveolin-2 may be a prognostic marker in OSCC.
Hämäläinen et al. (2019) (J Oral Pathol Med) (38)	Finland (University of Eastern Finland, Kuopio; University of Oulu; Kuopio University Hospital)	54 OLP biopsies (31 white/reticular/plaque, 19 red/atrophic-erosive) + 22 controls (healthy OM + normal mucosa from OSCC margins)	Histologically confirmed OLP (liquefaction degeneration of basal layer + band-like lymphocytic infiltrate)	Not specified	Claudin-1, Claudin-4, Claudin-7, E-cadherin, TWIST1, ZEB1	OLP (white vs. red subtypes) vs. normal mucosa	None (cross-sectional, no longitudinal follow-up)	Expression/localization of EMT-related proteins in epithelium and stroma	Claudin-7 ↑ in OLP stroma (both intensity and % positive cells, *p* < 0.001) E-cadherin ↓ in basal & intermediate epithelial layers in OLP (*p* < 0.001). TWIST1 & ZEB1 negative in epithelium; stromal positivity observed. TWIST1 + stromal cells fewer in OLP vs. controls (*p* < 0.001). ZEB1 stromal staining intensity weaker in OLP *(p* < 0.05)	Cross-sectional; small control group; interpretation of claudin-7 staining complicated by background signal; no clinical follow-up data.	Reduced Claudin-1, Claudin-4, and E-cadherin suggest early EMT-like junctional disruption in OLP → may contribute to barrier dysfunction & potential malignant transformation. TWIST1/ZEB1 appear stromal rather than epithelial, implying limited EMT involvement in OLP epithelium compared to dysplasia/OSCC
Tokozlu et al. (2024) (Pol J Pathol) (39)	Gazi University, Ankara, Turkey	58 FFPE cases: 19 OEH, 23 OED (9 LG, 14 HG), 16 OSCC (well/mod/poor)	Histologically confirmed OEH, OED, OSCC (2010–2015); WHO & binary grading	Not reported	EMT marker: Snail (IHC + RT-qPCR), E-cadherin (IHC)	CSC markers: CD133 (IHC + RT-qPCR), CD44 (IHC) Snail & CD133 mRNA (RT-qPCR) Correlation with inflammation score & histology	OEH vs. OED vs. OSCC	IHC (avidin-biotin method; antibodies for Snail, E-cadherin, CD133, CD44); RT-qPCR for Snail & CD133; scoring by H-score; SPSS non-parametric tests	Snail**:** IHC nuclear positivity in all groups; strongest in OSCC periphery & poorly differentiated areas. RT-qPCR: Snail ↑ in all OSCC, 85% OED, 82.5% OEH (not sig. between groups).	E-cadherin: Progressive loss from OEH (44%) → OED (74%) → OSCC (94%) (*p* = 0.006). CD133: IHC positive in 16.7% OEH, 26.1% OED, 53% OSCC (*p <* 0.05). RTqPCR posiTive only in few cases CD44: Positive in most samples across groups (no sig. difference). Correlations: CD133 immunodepression strongly correlated with inflammation score in OSCC (*r* = 0.661, *p* < 0.05)	Loss of E-cadherin and increase in CD133 are potential diagnostic/progression markers. Snail upregulation (esp. at invasive front) supports EMT role in dysplasia-carcinoma transition. CD133-inflammation link highlights microenvironment's role in OSCC progression.

*In vitro* studies							
Study (Author, Year)	Cell line(s)	Manipulation	Control group(s)	Assays/Methods	Molecular Targets	Analysis/Interpretation	Main Findings
Shang et al., (2023) (Oral Dis.) (27)	DOK (OLK-derived epithelial cells)	Lentiviral SNAI2 overexpression (Overexp) - Lentiviral *SNAI2* silencing (shRNA)	Negative control DOK cells UM1 (OSCC cell line, used as positive control for migration/invasion)	qPCR & Western blot (SNAI2 validation) CCK-8 assay (proliferation) Transwell migration & invasion assays Western blot for EMT markers	SNAI2 p-EMT markers: *LAMB3, LAMC2, PDPN* c-EMT markers: E-cadherin, N-cadherin, vimentin	* SNAI2* acts as a driver of partial EMT (p-EMT) in DOK cells. Overexpression promotes malignant transformation features (growth, invasion, EMT), while silencing reverses them. Supports role of *SNAI2* as therapeutic target in OLK progression	SNAI2 overexpression: ↑ proliferation, ↑ migration & invasion; ↑ LAMB3, LAMC2, PDPN, N-cadherin, vimentin; ↓ E-cadherin. *SNAI2* silencing: ↓ proliferation, ↓ migration & invasion; *↓* LAMB3, PDPN, N-cadherin, vimentin; ↑ E-cadherin. UM1 OSCC cells showed highest invasion/migration (positive control)
Xie et al. (2022) (Cell Death & Disease) (40)	HOK16E6E7 (immortalized oral keratinocytes), HaCat (human keratinocytes), 293 T (mechanistic assays)	Arecoline (20–60 µg/mL), dose- and time-dependent Overexpression: Flag-PA28*γ*, HA-MEK1, Myc-BRAF plasmids Knockdown: *si-PA28γ, si-MEK1, si-BRAF* Inhibitors: CI1040 *(MEK1* inhibitor)	Untreated epithelial cells (PBS) Vector controls (empty plasmids) siRNA negative control	CCK-8 assay (proliferation) Wound healing & Transwell (migration) Western blot/IF/IHC (PA28γ, MEK1, p-MEK1, p-ERK, EMT markers) Co-IP (PA28γ-MEK1-BRAF interaction) Ubiquitination assays	PA28γ MEK1, p-MEK1 ERK1/2, p-ERK BRAF EMT markers: E-cadherin, Vimentin, N-cadherin	Arecoline triggers EMT via the BRAF/PA28γ/MEK1 axis. BRAF protects PA28γ from degradation, enhancing MEK1/ERK activation. PA28γ is a potential therapeutic target in OSF progression toward OSCC.	Arecoline ↑ PA28γ*, p-*MEK1*,* p*-*ERK (dose- & time-dependent) Arecoline induced EMT: ↓ E-cadherin, ↑ Vimentin, N-cadherin; cells acquired spindle-like morphology & ↑ migration PA28γ interacted with MEK1, ↑ MEK1 phosphorylation; effect blocked by si-MEK1 or MEK inhibitor CI1040 BRAF interacted with PA28γ, stabilized *PA28*γ by inhibiting ubiquitination, promoting PA28γ/MEK1/ERK signaling
Zhao et al. (2015) (Int J Oncol) (18)	Primary human oral epithelial cells (HOEC*s*) - OSCC cell line: Cal27	Overexpression: Lentiviral transduction with hTERT *(*pCMV-hTERT*)* Knockdown: hTERT siRNA (3 sequences; siRNA#3 most effective) Serial passaging of hTERT-HOECs (>150 PDLs) Starvation for EGF in motility/invasion assays	HOECs infected with empty vector *(*pCMV) Cal27 with scrambled siRNA	Immunofluorescence & Confocal microscopy (E-cadherin, vimentin, β-catenin) Western blotting (*hTERT, p53,* E-cadherin, vimentin, Slug, Twist1, β-catenin, *p-GSK3*β) Wound healing assay (migration) Transwell invasion assay Cell morphology (phase-contrast microscopy)	hTERT EMT markers: E-cadherin (↓), Vimentin (↑), Slug (↑), Twist1 (↑) β-catenin (↑ nuclear translocation) GSK3β (↓ phosphorylation) p53 (↓ in hTERT-HOECs)	hTERT alone is sufficient to drive EMT in primary oral epithelial cells. EMT induction occurs via activation of the GSK-3β/β-catenin pathway. hTERT overexpression promotes motility and invasion, while silencing reverses EMT in OSCC cells. hTERT is a potential therapeutic target in OSCC progression**.**	hTERT overexpression in HOECs prolonged lifespan (>150 doublings vs. ∼20 in controls) and suppressed p53. HOECs with hTERT acquired EMT-like features: spindle morphology, ↓E-cadherin, ↑vimentin, Slug, Twist1. hTERT-HOECs showed ↑ migration and invasion vs. controls. hTERT activated Wnt/β-catenin pathway (cytoplasmic & nuclear β-catenin accumulation). Knockdown of *hTERT* in *Cal27* cells reversed EMT: ↑E-cadherin, ↓vimentin, Slug, Twist1, and inhibited β-catenin signaling
Chaw et al. (2012) (Oral Oncol) (31)	Human neoplastic oral keratinocytes (PE/CA-PJ15) in a Human Oral Mucosa Equivalent (HOME) organotypic culture on human dermis (63-day culture at air/liquid interface)	Long-term culture to mimic invasion of oral epithelium into connective tissue	Non-invasive epithelial layers within HOME culture (compared to invasive cells penetrating dermis)	Immunohistochemistry (IHC): E-cadherin, β-catenin, *APC*, Vimentin RT-PCR: mRNA levels of E-cadherin, Vimentin Confocal immunofluorescence microscopy (E-cadherin, Vimentin, APC, β-catenin localization)	E-cadherin (↓ in invasive cells) Vimentin (↑ in invasive cells) β-catenin (shift from membranous to cytoplasmic/nuclear) APC (heterogeneous, nuclear foci)	EMT occurs during malignant transformation: loss of epithelial marker (E-cadherin), gain of mesenchymal marker (Vimentin), and altered localization of β-catenin. Wnt/β-catenin pathway dysregulation implicated. APC upregulation may represent a compensatory but ineffective response to high β-catenin/Vimentin. *in vitro* organotypic invasion model validates EMT as a driver of OSCC invasion.	Invasive PE/CA-PJ15 cells within dermis lost E-cadherin expression and gained Vimentin expression, consistent with EMT. Non-invasive epithelial layers retained E-cadherin but showed little/no Vimentin. β-catenin showed cytoplasmic/nuclear localization in dysplasia & OSCC vs. membranous in normal. APC expression increased in OSCC and correlated positively with cytoplasmic/nuclear β-catenin and Vimentin. RT-PCR confirmed ↓E-cadherin (<1% of normal) and ↑Vimentin (∼3.7 × higher in OSCC vs. normal).
Kaneko et al. (2017) (Oral Oncol) (32)	OSCC cell lines: HSC-2, HSC-3, SQUU-A, SQUU-B, SQUU-BO *(Δ*Np63β-overexpression), SQUU-BC (vector control), SAS; keratinocyte line HaCaT	*Δ*Np63β overexpression (SQUU-BO) *Δ*Np63 knockdown (siRNA) *KLK6* knockdown (shRNA) PAR2 knockdown (siRNA) Exogenous recombinant *KLK6* (*rhKLK6*) protein treatment	Empty vector *(*SQUU-BC*)* Non-targeting siRNA/shRNA controls	RT-PCR & qPCR (KLK6, PAR1, PAR2, EMT markers) Western blot (E-cadherin, CK19, Vimentin, N-cadherin, Fibronectin, Snail, Slug, Twist, ZEB1/2, phospho-ERK, phospho-AKT) Morphology (phase contrast) WST-8 proliferation assay Wound healing assay (migration) Matrigel invasion assay (invasiveness)	*Δ*Np63 KLK6 PAR1, PAR2 EMT markers: E-cadherin, CK19, Vimentin, N-cadherin, Fibronectin, Snail, Slug, Twist, ZEB1/2 ERK, AKT phosphorylation	KLK6 has a dual role promotes malignant transformation (with ΔNp63β) via proliferation (*ERK* activation), but protects against EMT at invasive front (loss of *KLK6* promotes migration/invasion). EMT induction in OSCC linked to *KLK6* downregulation and *PAR1* upregulation when *ΔNp63β* is decreased. Suggests *KLK6-PAR2* axis suppresses EMT, while *PAR1* promotes EMT in OSCC progression.	ΔNp63β overexpression ↑ KLK6 & PAR2*, ↓ PAR1*; knockdown of ΔNp63 had the opposite effect. KLK6 knockdown → EMT phenotype: spindle-like morphology, ↓ E-cadherin/CK19, ↑ Vimentin, N-cadherin, Fibronectin, Snail, Slug, Twist, ZEB1 *.KLK*6 knockdown → ↓ proliferation, but ↑ migration and invasion. PAR2 knockdown → ↓ p-ERK, no change in p-AKT. Exogenous KLK6 (rhKLK6) restored E-cadherin, suppressed mesenchymal markers, reduced migration/invasion. ΔNp63β and KLK6 function mainly via KLK6-PAR2- ERK pathway.
Zheng et al. (2018) (Oncol Res) (34)	Primary human oral mucosal fibroblasts (hOMFs); SCC-9 (oral cancer); HEK293 *ex vivo***:** 10 OSF vs. 10 normal mucosal tissues	Arecoline (20–160 µg/mL) Tanshinone IIA (TSN) (5–50 µM) Arecoline + TSN co-treatment s*hRNA* knockdown (TP53, LSD1) LSD1 overexpression	Untreated cells Empty vector/non-targeting shRNA	Immunofluorescence (vimentin, keratin) MTT assay (proliferation) Western blot (E/N-cadherin, vimentin, p53, p21, Bax, PUMA, LSD1) mRNA microarray (GEO not listed) MSP (methylation-specific PCR) (TP53 promoter methylation) Luciferase reporter assay (p53 promoter) RIP & ChIP (LSD1 binding, H3K27me1, H3K4me2) - qRT-PCR	EMT markers: E-cadherin (↓ in EMT), N-cadherin, vimentin (↑ in EMT) Tumor suppressor: p53 & downstream (p21, Bax, PUMA) Epigenetic regulator: LSD1 Histone modifications: H3K27me1, H3K4me2	TSN epigenetically reactivates p53 by inhibiting LSD1-mediated demethylation → restores p53 function and suppresses EMT. TSN acts as an anti-fibrotic and anti-EMT agent in OSF fibroblasts exposed to arecoline. Provides mechanistic basis for developing TSN as a therapeutic strategy for OSF prevention and oral cancer transformation blockade.	Arecoline: ↑ proliferation, ↓ E-cadherin, ↑ N-cadherin & vimentin; ↓ p53, p21, Bax, *PUMA*; ↑ LSD1; ↑ TP53 promoter methylation. TSN: dose-dependent ↓ proliferation, ↑ p53 & downstream effectors, rescued E-cadherin, reduced N-cadherin & vimentin; reversed arecoline-induced EMT. LSD1 knockdown: ↑ p53 expression, epigenetic activation via ↑ H3K27me1 & H3K4me2 binding on TP53 promoter. TP53 knockdown: abolished *TSN*'s rescue effect, confirming dependence on p53 axis.
Liu et al. (2021) (J Cell Biochem) (35)	Leuk1 (OL cell line) Cal27 (OSCC cell line) HOK (normal keratinocytes, baseline)	Overexpression: pcDNA3.1-LOLA1 plasmid Knockdown: siRNA-LOLA1 Rescue: AKT inhibitor AZD5363	Empty vector (EV) Non-targeting siRNA Untreated cells	Microarray & qRT-PCR (lncRNA expression profiling) FISH (cellular localization) CCK-8 assay (viability) Flow cytometry (cell cycle, apoptosis) Transwell migration & invasion assays Western blot (E-cadherin, N-cadherin, Vimentin, AKT, p-AKT, GSK-3β, p-GSK-3β)	LOLA1 (lncRNA n386251) EMT markers: E-cadherin, Vimentin, N-cadherin AKT/GSK-3β pathway (p-AKT, p-GSK-3β)	LOLA1 drives EMT and invasiveness in OL cells via AKT/GSK-3β activation. Acts as a pro-oncogenic lncRNA in OL progression. Suggests LOLA1 could be both a predictive biomarker (for malignant transformation risk) and a therapeutic target in OPMD/OSCC	LOLA1 expression: stepwise ↑ from HOK → Leuk1 → Cal27; also ↑ in tissues from normal mucosa → OL (low/high risk) → OSCC. Overexpression in Leuk1: ↑ migration & invasion; ↓ E-cadherin; ↑ Vimentin & N-cadherin; ↑ p-AKT, p-GSK-3β. Knockdown in Leuk1: opposite effects (↓ invasion, ↑ E-cadherin, ↓ mesenchymal markers). AKT inhibitor (AZD5363): reversed LOLA1-induced migration, invasion, and EMT marker changes.
Wang et al. 2020 (Cell Death & Disease) (36)	OSCC cell lines: CAL27, SCC9, HN6, UM1 Normal control: HOKs HEK293T (for luciferase binding assays)	Overexpression: circEPSTI1 vector Knockdown: si-circEPSTI1 Rescue: miR-942-5p mimics/inhibitors Pathway inhibition: BEZ235 (dual PI3K/mTOR inhibitor)	Empty vector (EV) Non-targeting siRNA Vehicle control (DMSO)	circRNA microarray, RT-qPCR (expression) RNase R digestio*n* + Actinomycin D (circularity/stability) FISH (localization) CCK8, EdU, Colony formation (proliferation) Wound healing, Transwell (migration/invasion) Flow cytometry (cell cycle) RIP & RNA pull-down (miRNA binding) Dual luciferase assay (circRNA-miRNA & miRNA-mRNA interactions) Western blot, IHC, IF (EMT markers & pathway proteins)	circEPSTI1 miR-942-5p LTBP2 (ECM protein) EMT markers: E-cadherin, N-cadherin, Vimentin PI3 K/Akt/mTOR signaling (p-PI3K, p-Akt, p-mTOR)	circEPSTI1 acts as a pro-oncogenic circRNA in OSCC arising from OSF. It promotes EMT and malignant progression via the circEPSTI1 → miR-942-5p → LTBP2 → PI3 K/Akt/mTOR axis. Represents a potential biomarker and therapeutic target for OSCC with OSF.	circEPSTI1 expression: stepwise ↑ from NBM → OSF → OSCC; stable circular structure confirmed. Overexpression in CAL27/SCC9: ↑ proliferation, migration, invasion, colony formation; ↓ E-cadherin, ↑ N-cadherin & Vimentin (EMT). Knockdown: opposite effects; G1 arrest observed. Mechanism: circEPSTI1 sponges miR-942-5p → derepresses LTBP2 → activates PI3 K/Akt/mTOR. Rescue: miR-942-5p mimics/inhibitors reversed circEPSTI1 effects. - Pathway inhibition: BEZ235 blocked circEPSTI1-induced proliferation, invasion, and EMT changes.

*In vivo* studies											
CITATION	Animal model	Group size	Induction method	Control group	INTERVENTION	Monitoring	Sample processing	Histology/IHC	Molecular assays	Analysis	Findings
Shang et al., Oral Diseases (2023) (27)	C57BL/6 mice, 6 weeks old	Experimental: *n* = 17; Control: *n* = 3	Oral carcinogenesis induced by 4-nitroquinoline 1-oxide (4NQO) in drinking water (50 μg/mL) for 20 weeks	Received normal drinking water	Administration of 4-nitroquinoline 1-oxide (4NQO) in drinking water (50 μg/mL) for 20 weeks to induce the sequential progression from oral leukoplakia to OSCC in C57BL/6 mice	Weekly inspection of tongue lesions; OSCC defined when >20% lesions exceeded 2 mm diameter	Half lesions fixed in 4% paraformaldehyde (for H&E & IHC) Half frozen at −80 °C (for RNA-seq)	H&E staining for classification (normal, mild dysplasia, severe dysplasia, OSCC) IHC: *SNAI2* antibody (1:50), DAB detection, counterstain with hematoxylin	RNA-seq on selected 3 samples per group (for stability/reproducibility)	Expression of SNAI2 and p-EMT markers (LAMB3, LAMC2, PDPN*)* by RNA-seq + IHC Correlation analysis (Pearson r)	Gradual increase of *SNAI2, LAMB3, LAMC2* across stages (normal → OLK → OSCC) *PDPN* increased initially then decreased Stronger correlation of *SNAI2* with p-EMT markers (LAMB3, LAMC2) than with c-EMT markers (E-cadherin, vimentin)
Xie et al, (2022) (40)	BALB/c mice, female, 6 weeks old	Total *n* = 24; divided equally into 4 groups	Injections of areca nut into bilateral buccal mucosa every 2 days for 14 weeks using 29-gauge insulin syringe (50 μL per injection)	Normal drinking water (*n* = 3)	Chronic buccal injection of arecoline (1 mg/mL, 50 μL every 2 days for 14 weeks) in BALB/c mice to induce OSF (bleomycin and PBS as comparator groups)	Weekly inspection of tongue lesions; OSCC defined when lesions >2 mm and OSCC incidence >20%	Half of lesions fixed in 4% paraformaldehyde for H&E & IHC—Half frozen at −80 °C for RNA-seq	H&E used to classify: Normal (*n* = 3), mild dysplasia (*n* = 5), severe dysplasia (*n* = 8), OSCC (*n* = 4) *SNAI2* antibody (1:50)—Positive correlation with p-EMT markers (LAMB3, LAMC2)	RNA-seq on 3 randomly selected samples per group; correlation analysis (Pearson r)	IHC scoring based on staining intensity and proportion; collagen quantified with ImageJ	PBS: normal morphology, no fibrosis or EMT changes - Arecoline: induced OSF-like fibrosis and EMT (↑collagen, ↓blood vessels, ↑N-cadherin, ↑Vimentin, ↓E-cadherin)—Bleomycin: similar fibrotic/EMT changes validating the model—Mechanistically: *BRAF/PA28γ/MEK1* axis enhanced EMT and fibrosis progression *in vivo*
Zheng et al. (2018) (34)	BALB/c nude mice, male, 4–5 weeks old	5 mice per group	Subcutaneous xenograft: SCC-9 oral cancer cells (1×10⁶ in 200 μL**)** injected into the flank	Control (vehicle)	Arecoline (100 mg/kg/day by gavage) 3. Tanshinone *IIA* (150 mg/kg/day by gavage) 4. Arecoline + Tanshinone IIA (100 + 150 mg/kg/day)	Daily treatment for 7 weeks post-inoculation	Tumors harvested, paraffin-embedded, sectioned (4 μm) for H&E and IHC; protein lysates for Western blot	Ki-67 IHC: proliferation marker ↓ by TSN, ↑ by arecoline—EMT markers: Arecoline ↓E-cadherin, ↑N-cadherin & vimentin; TSN reversed this	Western blot for LSD1, p53, p21, puma, Bax, EMT markers	This study provided *in vivo* evidence that Tanshinone IIA suppresses arecoline-induced tumor growth and EMT. The mechanism involves epigenetic reactivation of p53 via inhibition of LSD1, restoring tumor suppressor signaling and reversing fibrosis/EMT. This highlights TSN as a potential therapeutic intervention for OSF and OSCC progression.	Arecoline promoted tumor growth and EMT in xenografts—TSN treatment significantly suppressed tumor growth and proliferation (Ki-67↓)—TSN reversed arecoline-induced changes in EMT markers (↓N-cadherin/vimentin, ↑E-cadherin)—Mechanism: *TSN* inhibited *LSD1*, restored p53 and downstream tumor suppressor molecules (p21, Bax, puma), and reduced hypermethylation of the TP53 promoter

The classification and synthesis of study types and outcomes were performed in accordance with the Cochrane Handbook for Systematic Reviews of Interventions (version 6.3) (42), ensuring consistency and methodological transparency in the identification, grouping, and interpretation of the included evidence. A detailed summary of each study's characteristics, including the specific OPMD subtype (leukoplakia, epithelial dysplasia, oral submucous fibrosis, or oral lichen planus) and comparative OSCC cohort, is provided in Table 2.

### 3.3 Quality and risk of bias assessment

The risk of bias assessment was performed according to the Cochrane Handbook for Systematic Reviews of Interventions (version 6.3), using validated domain-based tools appropriate for each study type. For *in vivo* studies, evaluation with the SYRCLE risk of bias tool revealed that all three studies exhibited a high overall risk of bias, primarily due to insufficient reporting of randomization, allocation concealment, and blinding procedures. Only one study (Xie and colleagues, 2022) (40) demonstrated low risk in specific domains related to outcome assessment. whereas the remaining studies (27, 34) lacked clarity in at least one methodological parameters.

**Figure 3:**
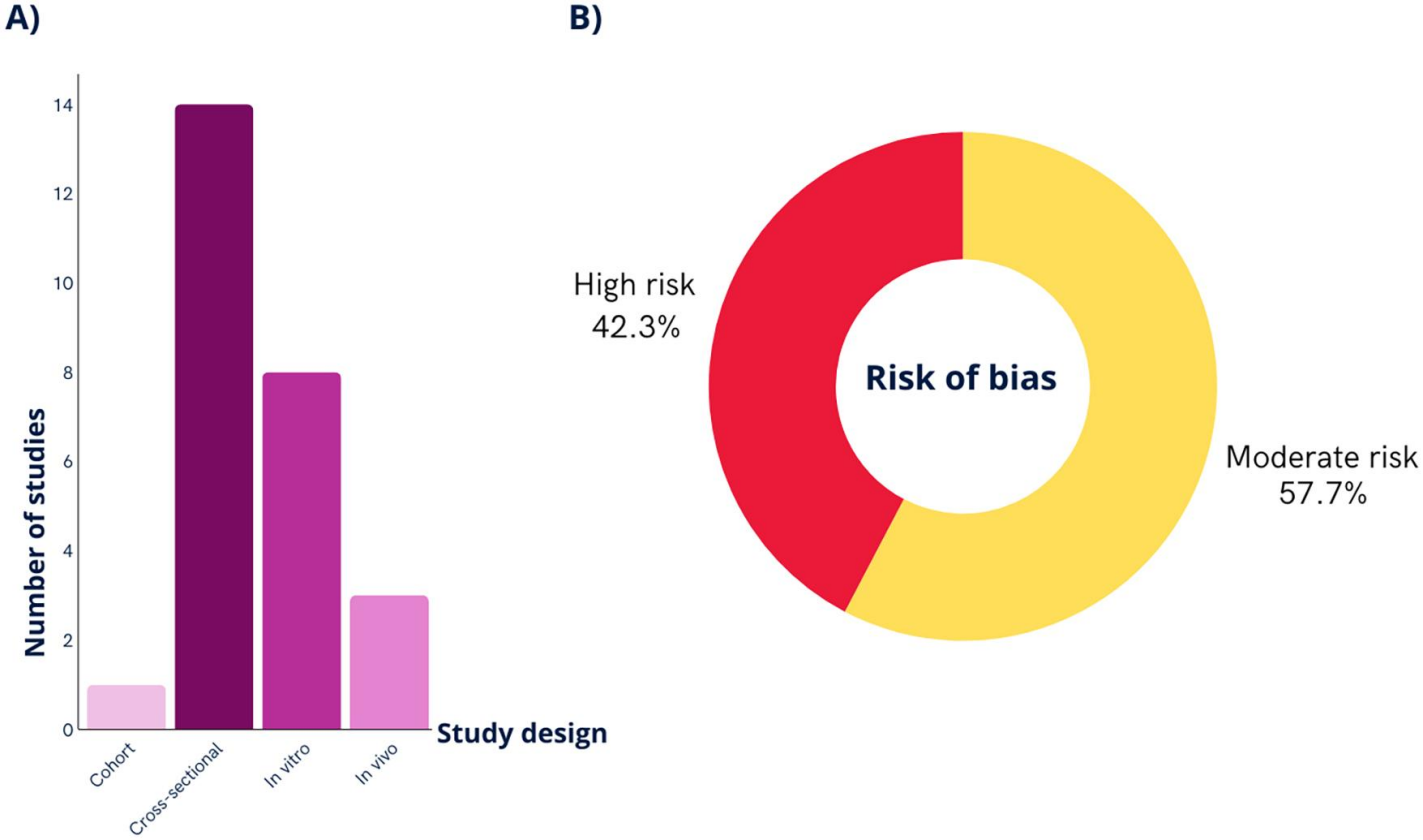
**(A)** Number of studies included plotted against each study design. **(B)** Quality and Risk of Bias Assessment.

Among the eight *in vitro* studies, assessment using the QUIN tool showed that the majority had a moderate risk of bias. Common methodological limitations included inadequate description of replication strategy, lack of randomization, and limited information regarding statistical power or reproducibility. Nevertheless, all *in vitro* studies clearly reported experimental conditions and outcome measures, reducing detection bias. The single cohort study demonstrated an overall moderate risk of bias, with unclear reporting in selection and confounding domains but acceptable performance in exposure measurement and outcome assessment. Regarding the cross-sectional human tissue-based studies (*n* = 5), evaluated using the JBI Critical Appraisal Checklist, most were classified as moderate risk of bias (Supplementary Tables 1–4). The main sources of bias included unclear participant selection criteria, variability in YAP/EMT immunohistochemical scoring methods, and lack of blinding during assessment. Overall, the majority of the included studies presented a moderate risk of bias, reflecting acceptable methodological quality but highlighting the need for more standardized reporting, randomization, and blinding in future investigations. Inter-reviewer agreement was high (*κ* = 0.87), indicating consistent evaluation across all domains (Figure 3).

### 3.4 Data synthesis and thematic findings

Across *in vivo*, *in vitro*, cohort, and cross-sectional evidence, EMT features were consistently observed along the OPMD to OSCC continuum. Hallmarks included loss of epithelial adhesion (decreased E-cadherin) with concomitant gain of mesenchymal programs (increased N-cadherin, increased vimentin) and increased motility/invasion. Multiple studies further implicated dysregulation of Hippo-YAP signaling and its crosstalk with MAPK/ERK, PI3K/Akt/mTOR, Wnt/β-catenin, and p53/epigenetic axes in promoting malignant transformation, involving EMT transcription factors such as SNAI1, SNAI2, TWIST1, and ZEB1.

1. EMT programs are activated early and intensify with progression (43). Human tissue studies showed stepwise EMT marker shifts from normal mucosa to OPMDs and OSCC characterized by progressively decreased E-cadherin, increased vimentin and EMT regulators such as Snail/Twist). In fact, through a so-called tumor-front phenomena, at the invasive front of OSCC, small clusters of dedifferentiated tumor cells known as tumor budding represent a key histopathological feature associated with EMT; these buds often exhibit nuclear positivity for EMT transcription factors such as Snail and Twist for acquisition of mesenchymal traits (33). Clinically, tumor budding combined with Snail/Twist expression correlates with lymph-node metastasis and poorer overall survival (33). In oral submucous fibrosis (OSF), a high-risk OPMD, stromal activation characterized by the presence of cells expressing alpha smooth muscle actin (*α*-SMA), a known marker for myofibroblasts and pericytes, and increase in the angiogenesis marker Factor VIII was accompanied by elevated expression of hypoxia-inducible factor 1-alpha (HIF-1*α*) (16, 28), reinforcing the role of hypoxia and oxidative stress in driving EMT and angiogenesis. These microenvironmental changes, together with extracellular matrix remodeling, create mechanical cues that activate YAP/TAZ signaling, supporting a hypoxia/oxidative-stress-driven EMT/angiogenesis axis in oral carcinogenesis (16).
2. Hippo-YAP dysregulation aligns with dysplasia severity and OSCC aggressiveness (23).
  Two IHC-based analyses (23, 30), combining a total of 340 cases demonstrated rising YAP expression from normal to OED and OSCC, with higher nuclear YAP associated with larger tumors, advanced stage, and poorer differentiation. An inverse relationship with *PARK2* suggested loss of a potential YAP-modulating tumor suppressor. These findings position YAP as an integrative node coupling epithelial integrity loss to invasive behavior.
3. Mechanistic pathways converging on EMT (11). Apart from Hippo–YAP signaling, multiple oncogenic pathways converge to amplify EMT programs, and create a complex regulatory network to promote invasion, metastasis, and therapy resistance. MAPK/ERK axis (arecoline to BRAF/PA28*γ*/MEK1): Chronic exposure to arecoline, an alkaloid found in areca nut (betel nut), is commonly chewed in many Asian countries and is a major etiological factor for OSF (44), and was shown to induce EMT by decreasing E-cadherin and increasing N-cadherin and vimentin expression via stabilization of PA28*γ* by *BRAF* and activation of MEK1/ERK; pharmacologic or siRNA blockade attenuated EMT (40). Introduce the importance of this axis Epigenetic/p53 axis (LSD1-p53): Tanshinone IIA (TSN), a bioactive compound extracted from *Salvia miltiorrhiza* (commonly known as Danshen, a traditional Chinese medicinal herb) has been utilized in pharmacotherapy and was shown to suppress EMT and tumor growth, supporting translational plausibility (34). TSN reversed arecoline-induced EMT by inhibiting *LSD1*, reactivating p53 programs (increased p21, Bax, PUMA) and restoring E-cadherin; TP53 knockdown abrogated TSN's rescue, pinpointing p53 dependence (34).

The Wnt/β-catenin signaling pathway plays a pivotal role in maintaining epithelial integrity and regulating cell fate. Dysregulation of this axis is strongly linked to EMT and cancer progression, as nuclear translocation of β-catenin activates transcriptional programs that drive invasion and stemness (18). Wnt/β-catenin axis (*hTERT*): In oral carcinogenesis, *hTERT* overexpression alone was sufficient to drive EMT in primary oral epithelial cells, with β-catenin nuclear translocation and enhanced migration/invasion; *hTERT* knockdown reversed EMT in OSCC cells (18).

The protease-activated receptor (PAR) signaling network plays a critical role in tumor invasion and EMT by modulating extracellular proteolysis and cell–matrix interactions. In OSCC, dysregulation of kallikrein-related peptidase 6 (*KLK6*) and PAR receptors at the invasive front influences EMT dynamics and metastatic potential (32). Protease/PAR signaling (ΔNp63-KLK6-PAR): *KLK6* loss (with ΔNp63β reduction) promoted EMT, migration, and invasion, whereas exogenous *KLK6* dampened EMT and restored E-cadherin, implicating a KLK6-PAR2-ERK suppressive axis vs. PAR1-driven EMT at the invasive front (32). PI3K/Akt/mTOR axis (circEPSTI1/miR-942-5p/LTBP2): circEPSTI1 upregulation (with reciprocal miR-942-5p loss and LTBP2 gain) activated PI3K/Akt/mTOR, promoting EMT and poor outcomes; pathway inhibition (BEZ235) mitigated the EMT phenotype (36).

4. Putative biomarkers for progression risk and prognosis (27). Among EMT transcription factors, *SNAI* family genes (particularly *SNAI2*) have emerged as key regulators of EMT programs, being recognized as predictive biomarkers of malignant transformation risk in OPMD. *SNAI2* (p-EMT regulator): *in vivo* and tissue data showed increasing *SNAI2* across stages and stronger correlation with p-EMT markers (LAMB3/LAMC2) than with c-EMT markers (27). In an oral leukoplakia cohort, high *SNAI2* predicted malignant transformation (HR ≈ 2.5; *p* ≈ 0.03) and improved model discrimination (27).

KLK6 at the invasive front: Low KLK6 predicted worse outcomes (HR ≈ 4.0; *p* < 0.001), with higher recurrence/metastasis, consistent with a protective role against EMT at the front (32). lncRNA LOLA1: In OPMD cohorts, malignant transformation occurred in ∼28.6% of LOLA1-high vs. ∼2.2% of LOLA1-low cases (*p* ≈ 0.001), nominating LOLA1 as a stratification biomarker (35). circEPSTI1 triad (circEPSTI1-miR-942-5p-LTBP2): Stepwise increase from normal to OSF and to OSCC; high circEPSTI1/low miR-942-5p/high LTBP2 associated with advanced stage and poorer survival. The combined evaluation of YAP and PARK2 (YAP/PARK2 duplex): Higher YAP with lower PARK2 associated with adverse clinicopathologic features, such as larger tumor size (T3–T4), advanced clinical stage (III–IV), and poor histological differentiation, suggesting that loss of PARK2 may permit YAP-driven EMT and aggressive behavior; survival trends were consistent but not statistically significant in short follow-up (30, 36).

**Figure 4:**
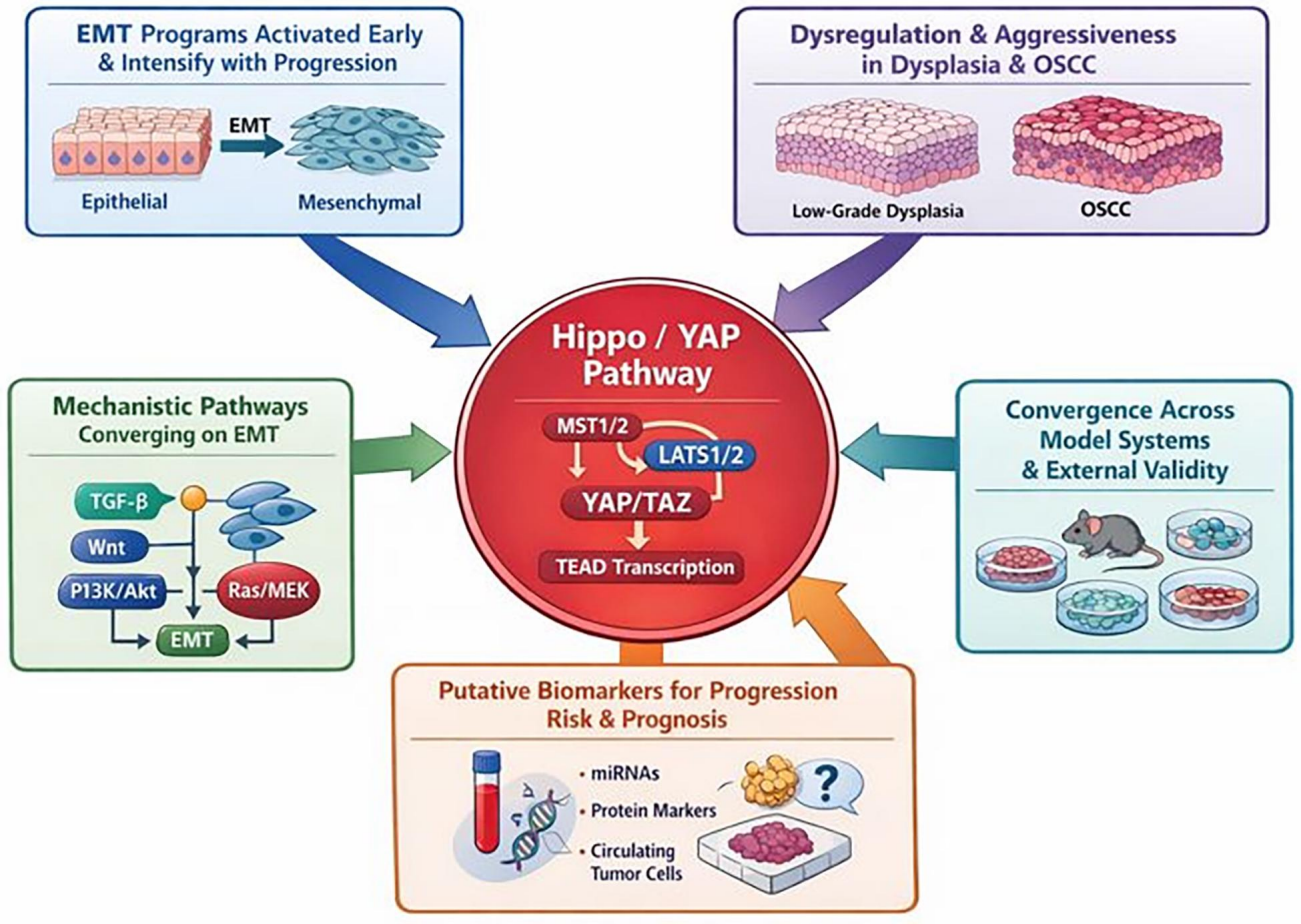
Conceptual summary of the Hippo-YAP pathway as a central integrative node in OPMD-to-OSCC progression. This schematic is based on the extracted data from the studies summarized in Table 2 and illustrates how dysregulation of Hippo-YAP signaling links five key thematic findings of the review. (1) EMT programs are activated early in oral potentially malignant disorders and intensify with histopathological progression toward OSCC. (2) Increasing Hippo–YAP dysregulation, particularly nuclear YAP/TAZ activity, aligns with dysplasia severity and OSCC aggressiveness. (3) Multiple mechanistic pathways, including MAPK/ERK, PI3K/Akt/mTOR, Wnt/β-catenin, epigenetic/p53, and protease/PAR signaling, converge on EMT and interface with Hippo-YAP signaling. (4) EMT and Hippo-YAP-related molecules emerge as putative biomarkers for malignant transformation risk and prognosis. (5) Convergent evidence across human tissues, *in vitro* systems, and *in vivo* models supports the external validity of Hippo-YAP-centered EMT regulation in oral carcinogenesis.

5. Convergence across model systems and external validity (31). *In vivo* 4NQO studies refer to chemical carcinogenesis models where 4-nitroquinoline-1-oxide (4NQO), a DNA-damaging agent, is administered (usually in drinking water) to rodents to mimic the stepwise progression from normal oral mucosa to leukoplakia, dysplasia, and OSCC; this model closely recapitulates human oral carcinogenesis, making it widely used for mechanistic and chemoprevention research (45). Chemical (4NQO) and arecoline-based models reproduced EMT activation and captured stage-linked changes in EMT/p-EMT effectors (27). A conceptual summary of the Hippo-YAP pathway as a central integrative node in OPMD-to-OSCC progression is build upon the abovementioned five keypoints and depicted in Figure 4.

Regarding heterogeneity, risk of bias, and certainty, most *in vitro* studies carried moderate risk of bias (QUIN), whereas *in vivo* studies were high risk overall (SYRCLE) due to under-reported randomization/blinding, and human observational studies were moderate risk (JBI) (Supplementary Tables 1–4). Heterogeneity stemmed from variable scoring of nuclear YAP/EMT markers, diverse exposure models (arecoline vs. 4NQO), and limited longitudinal follow-up. Nonetheless, the direction of effect (EMT activation and Hippo-YAP involvement in OPMD to OSCC) was consistent across platforms.

## 4 Discussion

The present systematic review synthesized data from human tissue studies, *in vitro* and *in vivo* models, and retrospective cohorts, with a particular focus on the interplay between the Hippo-YAP signaling pathway and EMT during the progression of OPMDs to OSCC. Interestingly, classical EMT markers demonstrated reproducible trends across studies. E-cadherin was reduced in OED, OSCC, and OSF-associated carcinoma, with lower levels correlating with nodal metastasis and poorer prognosis (23, 30). Conversely, vimentin expression progressively increased with histological severity, reinforcing its role as a marker of invasive potential (29, 31, 39).

EMT represents the morphological and functional expression of the molecular changes initiated by Hippo-YAP dysregulation, bridging intracellular signaling with tumor cell behavior. The Hippo-YAP signaling pathway plays a crucial role in the regulation of EMT, a process fundamental to tumor invasion and metastasis. When Hippo signaling is inactive, the downstream effectors YAP/TAZ become dephosphorylated and translocate into the nucleus, where they interact with TEAD transcription factors to activate EMT-promoting genes such as *SNAI1/2*, *ZEB1*, and *TWIST1* (8, 15). These transcriptional programs lead to repression of epithelial markers like E-cadherin (CDH1) and induction of mesenchymal markers including vimentin (VIM) and N-cadherin (CDH2), promoting cytoskeletal remodeling, loss of cell-cell adhesion, and increased motility (8, 18). Conversely, activation of the upstream Hippo kinases MST1/2 and LATS1/2 phosphorylates YAP, leading to its cytoplasmic retention and degradation, thereby preventing EMT initiation (15). Collectively, these findings indicate that dysregulated Hippo-YAP signaling functions as a master regulator of EMT, linking aberrant mechano-transduction and transcriptional reprogramming to malignant transformation in OSCC.

As mentioned, across diverse experimental and clinical studies, consistently, and to some extent expected, alterations in canonical EMT markers (loss of E-cadherin, gain of vimentin and N-cadherin, nuclear translocation of β-catenin) were observed (28, 29, 31, 39). Interestingly though, such variations reliably appeared alongside emerging evidence that Hippo-YAP dysfunction acts as a master regulator of these processes (23, 30). For instance, studies have demonstrated that YAP nuclear accumulation increases from normal oral mucosa to dysplasia and OSCC, coinciding with loss of E-cadherin and gain of vimentin in epithelial cells, consistent with YAP-mediated EMT during malignant transformation (23). Similarly, Zhang and colleagues (22) reported migration and invasion of oral keratinocytes through Snail and Slug protein activation, downstream of YAP overexpression, confirming YAP's role as a transcriptional driver of EMT in OSCC (46).

Ideally, mechanisms identified in cellular models should reproducibly manifest *in vivo* and ultimately enable patient risk prediction. Many of the mechanisms highlighted in this review remain at the level of first-line, evidence-based research (*in vitro*). However, they represent promising foundations for future clinical discoveries. For instance, *SNAI2* drives a p-EMT program in dysplastic oral keratinocytes (DOK cells) by promoting an increase in *LAMB3/LAMC2/PDPN* expression, decrease of E-cadherin and increase of migration/invasion, mirroring a stepwise SNAI2-LAMB3/LAMC2 rise from normal to oral leukoplakia to OSCC in 4NQO mice. Such increased expression of *SNAI2*, was associated with a two-fold increase in the malignant transformation risk of oral leukoplakias (HR = 2.50, 95% CI 1.08–5.79) (27). Likewise, arecoline triggers EMT via BRAF/PA28γ/MEK1 to *ERK* in keratinocytes and induces OSF-like fibrosis and EMT *in vivo.* Interestingly, TSN reverses arecoline-driven EMT and growth in xenografts by inhibiting *LSD1* and reactivating p53 (34, 40). In patients, the circEPSTI1- miR-942-5p- LTBP2- PI3K/Akt/mTOR axis tracks with advanced tumor stage and worse overall survival/progression-free survival (OS/PFS), and is pharmacologically reversible with BEZ235 *in vitro* and *in vivo* (41).

### 4.1 EMT as the phenotypic hallmark of progression

β-catenin exhibited a shift from membranous to cytoplasmic and nuclear localization, consistent with canonical Wnt pathway activation and transcriptional reprogramming during EMT (28, 31). Across lesions, the EMT readout scales were established with severity: E-cadherin loss increased from oral epithelial hyperplasia (OEH) to OSCC (44%–94%) (*p* = 0.006) (39). In an organotypic model, invasive cells showed E-cadherin mRNA <1% of levels in normal oral epithelial cells, vimentin around 3.7-fold higher, and β-catenin shifted from membranous to cytoplasmic/nuclear shift (31). In OSF progressing to OSCC, E-cadherin localization moved from membranous (80%) to cytoplasmic (40%), vimentin expression rose from 18.0 to 22.2, and E-cadherin intensity showed a moderate negative correlation with vimentin (Spearman *ρ* ≈ −0.54) (29). In OSF and OSCC tissues, hypoxia and oxidative stress were prominent microenvironmental drivers of EMT. Microenvironmental stress paralleled these shifts: HIF-1α and 4-HNE tightly correlated (*r* = 0.98) with β-catenin cytoplasmic/nuclear diffusion and matrix remodeling, reinforcing EMT as the phenotypic endpoint of upstream signaling (28). These observations collectively confirm that EMT intensifies with lesion severity and is driven by β-catenin nuclear translocation, E-cadherin loss, vimentin gain, and microenvironmental stress.

### 4.2 Hippo-YAP signaling as a driver of EMT

Multiple studies demonstrated that YAP overexpression occurs early in high-grade dysplasia and carcinoma *in situ* and remains elevated in invasive OSCC (23, 30). High YAP expression consistently correlated with advanced clinical stage and poorer survival outcomes, underscoring its potential as a prognostic biomarker (23, 30). Parallel increases in *Δ*Np63, a specific isoform of the p63 protein that is part of the p53 family of transcription factors, further highlight the role of epithelial stemness and Hippo pathway inactivation in promoting transformation (23). Taken together, these results indicate that Hippo-YAP dysregulation functions as a molecular “switch” that accelerates the progression of premalignant lesions into malignancy by enforcing EMT programs (23).

Issue data show YAP rises stepwise from normal oral mucosa to OSCC (*p* = 0.025) and associates with larger tumors (T3-T4, *p* = 0.034), advanced stage (III–IV, *p* = 0.034), and poorer differentiation (*p* = 0.001); *PARK2* decreases (*p* = 0.005) and inversely correlates with YAP (*r* = −0.381) (30). A larger series confirmed higher YAP/ΔNp63 in high-grade oral epithelial dysplasia/carcinoma *in situ* (OED/CIS) and in less-differentiated OSCC (23). Functionally connected drivers converge on EMT: *hTERT* is sufficient to induce EMT and β-catenin nuclear accumulation in primary oral epithelial cells, with reversal upon *hTERT* knockdown (18). ΔNp63/KLK6 balance modulates invasion, and low *KLK6* at the invasive front links to poor outcomes (32).

In fact, functional assays in OPMD and OSCC cell lines demonstrated correlation between key pathway nodes and EMT phenotypes. For example, *hTERT* overexpression in primary oral keratinocytes induced spindle morphology, E-cadherin loss, vimentin gain, and enhanced migration/invasion, while siRNA knockdown reversed these changes (18). Arecoline exposure stabilized *PA28*γ and activated MEK1/ERK signaling, driving EMT (40). *MEK* inhibition (CI1040) blocked this cascade (40). Similarly, *LSD1* overexpression suppressed *TP53* and promoted EMT (34), whereas TSN restored p53 and epithelial markers. ΔNp63β knockdown reduced *KLK6* and triggered EMT, which was reversed by recombinant KLK6 (32), and circEPSTI1 overexpression activated PI3K/Akt/mTOR signaling to promote EMT, with BEZ235 reversing these effects (36). Collectively, these gain/loss-of-function experiments in OPMD/OSCC lines causally linked pathway nodes (hTERT, PA28γ/MEK1, LSD1/p53, ΔNp63/KLK6, circEPSTI1) to EMT phenotypes and invasive behavior (18, 32, 34, 36, 40). Together these data support Hippo-YAP and its partners as upstream enforcers of EMT programs.

### 4.3 Crosstalk between Hippo-YAP and EMT pathways

Recent evidence underscores that Hippo-YAP and EMT are not isolated processes but interdependent and mutually reinforcing. YAP interacts with canonical EMT regulators such as Snail, Twist, and ZEB1 to repress epithelial adhesion molecules and induce mesenchymal programs (8). Crosstalk with Wnt/β-catenin signaling has been documented, whereby YAP activation coincides with nuclear β-catenin translocation and enhanced transcription of EMT-related genes in both tissue and *in vitro* models (18, 31). Furthermore, Hippo-YAP integrates with PI3K/Akt/mTOR (35, 41) and MAPK/ERK (40) pathways, amplifying proliferative and invasive signals. This integration suggests that Hippo-YAP functions as a central hub orchestrating EMT through multiple downstream axes, thereby accelerating the malignant conversion of OPMDs (23, 30).

Non-Hippo inputs feed EMT circuits that interface with YAP outputs (11). For instance, arecoline stabilized *PA28*γ (via *BRAF*), increased p-MEK1/p-ERK, and induced EMT; MEK inhibition (CI1040) blocked the cascade (31). The circEPSTI1 axis sponged miR-942-5p to derepress *LTBP2*, activating PI3K/Akt/mTOR and producing EMT; BEZ235 reversed these phenotypes and the axis associated with advanced T/TNM and worse OS/PFS (36). Convergence with Wnt signaling is supported by β-catenin nuclear shift in tissues (28, 31) and by *hTERT*-driven β-catenin activation in primary oral epithelial cells (18). Partial EMT markers (LAMB3/LAMC2/PDPN) partnered with *SNAI2* across normal mucosa to OPMD and to OSCC *in vivo*, linking EMT transcriptional programs to histologic progression (27). These interacting axes suggest that Hippo-YAP is closely interconnected with EMT regulation through ERK, PI3K/mTOR, and Wnt arms.

### 4.4 Prognostic significance of Hippo-YAP/EMT activation

The clinical implications of these molecular alterations are substantial. EMT markers such as tumor budding, combined with Snail or Twist positivity, strongly correlated with lymph node metastasis and reduced overall survival (33, 39). Similarly, high expression of YAP, hTERT, and circEPSTI1, as well as loss of KLK6 at the invasive front, were associated with worse prognosis and shorter disease-free survival (18, 30, 32, 36). These findings confirm that EMT-related markers, particularly when integrated with Hippo-YAP status, can serve as valuable biomarkers for risk stratification of OPMDs. Identifying patients with high-risk EMT/YAP signatures could enable closer surveillance, early intervention, or enrollment into chemopreventive strategies.

Quantifiable risk signals emerge across datasets. High *SNAI2* in OPMD were linked to an increased malignant transformation (HR = 2.50, 95%CI 1.08–5.79) (27). In a leukoplakia cohort, high expression of the lncRNA LOLA1 was associated with malignant transformation in 28.6% of LOLA1-high vs. 2.2% of LOLA1-low cases (*p* = 0.001) (35)). In established OSCC, tumor budding ≥3 associated with lymph node metastasis (*p* = 0.001) and worse OS (*p* = 0.002), co-occurring with Snail/Twist positivity (33). At the invasive front, low KLK6 quadrupled cause-specific death risk (HR = 4.01; 95%CI 1.79–9.59) (32). Recent studies have identified molecular signatures that link EMT activation and Hippo-YAP dysregulation with clinical outcomes in OSCC and OPMDs (11). Metabolic-EMT coupling (higher caveolin-2/FABP5, lower E-cadherin) predicted recurrence and adverse outcomes in OSCC (27). High circEPSTI1/low miR-942-5p/high LTBP2 expression portended worse OS and PFS in OSF-associated OSCC (36). Although YAP/PARK2 survival trends did not reach statistical significance in one cohort, the direction of effect was consistent with larger series and with the biological model of YAP activation and PARK2 loss (23, 30). Collectively, these findings indicate that diverse molecular signatures, from EMT regulators and noncoding RNAs to Hippo-YAP components, carry measurable prognostic value, offering opportunities for risk stratification and targeted surveillance in OPMDs and OSCC.

### 4.5 Therapeutic perspectives

A growing body of preclinical work indicates that EMT induced by Hippo-YAP activation is potentially reversible (47). Pharmacological inhibition of PI3K/mTOR (BEZ235) suppressed circEPSTI1-driven EMT in OSCC cells (36). *LSD1* inhibition restored TP53 expression and blocked arecoline-induced EMT in OSF fibroblasts and co-culture models, while also correcting TP53 promoter hypermethylation (34). MEK1/BRAF pathway inhibition reversed PA28γ-mediated EMT and fibrosis in arecoline-treated keratinocytes and *in vivo* models (40). Similarly, genetic silencing of *hTERT* or *SNAI2* restored E-cadherin expression and reduced invasion (18, 27). These results suggest that targeting Hippo-YAP and its downstream EMT effectors may represent a viable strategy not only for treating established OSCC but also for preventing malignant progression in high-risk premalignant lesions.

Pharmacologic interventions demonstrated that EMT-related pathways can be reversed at multiple molecular targets (48). *In vivo*, TSN curtailed arecoline-augmented xenograft growth, lowered Ki-67, restored E-cadherin, reduced N-cadherin/vimentin, inhibited *LSD1*, and reactivated *TP53* to p21/Bax/PUMA while decreasing *TP53* promoter hypermethylation (34). *In vitro*/*in vivo* studies showed that CI1040 blocked arecoline-induced PA28γ-MEK1/ERK activation and EMT (40), while BEZ235 reversed circEPSTI1-driven EMT and proliferation (41). *SNAI2* silencing (27) and *hTERT* knockdown (18) restored epithelial markers and reduced invasion. These convergent reversals nominate *MEK*, PI3K/mTOR, *LSD1*, hTERT/SNAI2 as testable chemopreventive targets in high-risk OPMD. Together, these findings demonstrate that EMT-inducing pathways are not fixed but can be pharmacologically modulated at multiple molecular nodes, offering promising targets for chemoprevention in high-risk OPMDs.

### 4.6 Risk of bias and limitations

Despite consistent findings, several methodological limitations restrict the strength of the conclusions. Risk of bias assessments indicated that most *in vivo* studies were at high risk, with poor reporting of randomization, allocation concealment, and blinding (SYRCLE tool) (27, 34, 40). *In vitro* studies generally showed moderate-to-high bias, particularly regarding replication and blinding (QUIN tool) (18, 35). Human studies, predominantly cross-sectional, also carried moderate risks due to heterogeneity in IHC scoring, small sample sizes, and limited control of confounders. These limitations highlight the urgent need for longitudinal, multi-center studies employing standardized protocols for biomarker evaluation and outcome reporting (29, 31, 37, 39). Some key survival results lacked multivariable adjustment such as tumor budding and KLK6 studies) (32, 33) and in leukoplakia cohorts, relatively few transformation events (5 of 60 OPMD cases progressing to OSCC) reduced the precision of effect estimates (35). These limitations highlight the urgent need for longitudinal, multi-center studies employing standardized protocols for biomarker evaluation and outcome reporting.

Further design constraints reduce the strength of inference, including small animal cohorts with short treatment windows and incomplete reporting of randomization and blinding (34), as well as reliance on single-cell line models, such as Cal27 line for *hTERT* knockdown experiments and Leuk1 line for *LOLA1* overexpression and silencing studies, with limited rescue conditions (18, 35), cross-sectional tissue series with heterogeneous IHC scoring methods (28, 29, 37, 39), and survival analyses without full multivariate adjustment (32, 33). Collectively, these factors argue for larger, standardized, prospective designs to validate Hippo-YAP/EMT markers for clinical use.

### 4.7 Future directions

Future investigations should prioritize:

– Large, prospective cohorts of OPMD patients to validate Hippo-YAP/EMT marker panels for malignant risk prediction.
– Standardized immunohistochemical scoring systems for YAP and EMT markers to enhance reproducibility.
– Integration of multi-omics platforms (transcriptomics, methylation, proteomics, circRNA analysis) to construct comprehensive EMT-Hippo regulatory networks.
– Development of risk stratification algorithms combining histopathology, molecular EMT markers, and Hippo-YAP signatures.
– Translational studies assessing the efficacy of EMT-targeted therapeutics in chemoprevention and treatment of OPMDs and OSCC

## 5 Conclusion

Evidence compiled in this review is consistent with the notion that Hippo-YAP dysregulation and EMT activation may jointly contribute to the malignant transformation of oral premalignant lesions. YAP nuclear accumulation not only disrupts growth control but also initiates transcriptional cascades that enforce EMT, while EMT provides the cellular plasticity required for invasion, migration, and metastasis. Recognizing the synergistic role of these pathways strengthens our mechanistic understanding of oral carcinogenesis and underscores their promise as biomarkers for early detection and prognosis as well as targets for therapeutic intervention and chemoprevention.

## Data Availability

The raw data supporting the conclusions of this article will be made available by the authors, without undue reservation.
