# Supplementary material for: Crosstalk of the Hippo/YAP pathway in the progression of oral potentially malignant disorders to oral squamous cell carcinoma: a systematic review
Source: Front Oral Health. 2026 May 25;7:1808680. doi: 10.3389/froh.2026.1808680 (PMC13243288; doi:10.3389/froh.2026.1808680)
Supplement: Supplementary file 1 [file Table1.docx]

**Supplementary Table 1. In vivo study (SYRCLE RoB tool).**

| study | Domain 1 | Domain 2 | Domain 3 | Domain 4 | Domain 5 | Domain 6 | Domain 7 | Domain 8 | Domain 9 | Domain 10 | overall |
| --- | --- | --- | --- | --- | --- | --- | --- | --- | --- | --- | --- |
| Emt 1  Shang et al., 2023 | High/unclear | unclear | unclear | unclear | unclear | High/unclear | unclear | unclear | unclear | high | high |
| Emt8  Xie C et al.2022 | High/unclear | unclear | unclear | unclear | unclear | Hight/unclear | unclear | low | low | moderate | high |
| Emt32  Zheng et al.2018 | high | moderate | high | High | high | high | low | moderate | moderate | moderate | high |

**Supplementary Table 2. In vitro studies (QUIN risk of bias tool).**

| Study | Domain1 | Domain 2 | Domain3 | Domain4 | Domain5 | Domain 6 | Domain 7 | Domain8 | Domain 9 | overall |
| --- | --- | --- | --- | --- | --- | --- | --- | --- | --- | --- |
| Emt1 | low | Moderate | high | high | Moderate | low | low | low | moderate | Moderate |
| Emt8 | low | moderate | High | high | moderate | low | low | low | moderate | Moderate |
| Emt14  Zhao et al.2015 | low | low | Moderate/high | high | Low/moderate | high | Low/moderate | low | moderate | Moderate/high |
| Emt21  Chaw SY et al,2012; | low | moderate | high | high | moderate | low | low | low | moderate | Moderate/high |
| Emt29  Kaneko N et al.2017 | low | moderate | high | high | moderate | low | low | low | moderate | moderate |
| Emt32  Zheng et al.2018 | low | moderate | high | high | moderate | low | low | low | moderate | Moderate-high |
| Emt46 | low | moderate | high | high | moderate | low | low | low | moderate | moderate |
| Emt53 | low | low | high | high | moderate | low | low | low | moderate | Moderate-high |

**Supplementary Table 3. JBI Critical Appraisal Checklist for Cohort Studies.**

| Study ID | Domain1 | Domain 2 | Domain 3 | Domain 4 | Domain 5 | Domain 6 | overall |
| --- | --- | --- | --- | --- | --- | --- | --- |
| Emt1 | unclear | unclear | Low/moderate | low | Moderate/high | low | moderate |

**Supplementary Table 4. JBI Critical Appraisal Checklist for Analytical Cross-Sectional Studies.**

| Study ID | Domain1 | Domain2 | Domain 3 | Domain 4 | Domain5 | Domain 6 | Domain 7 | Domain 8 | Domain 9 | Domain 10 | overall |
| --- | --- | --- | --- | --- | --- | --- | --- | --- | --- | --- | --- |
| Emt12 | low | Low/moderate | low | low | Moderate-high | high | Low/moderate | low | moderate | Moderate | Moderate |
| Emt14 | low | Low/moderate | low | low | moderate | high | Low/moderate | low | moderate | moderate | moderate |
| Emt19 | low | moderate | low | low | moderate | high | moderate | low | moderate | moderate | moderate |
| Emt 20 | low | Moderate | low | low | moderate | high | moderate | low | moderate | moderate | moderate |
| Emt21 | low | moderate | low | low | moderate | high | moderate | low | Moderate | moderate | moderate |
| Emt 29 | low | moderate | low | low | moderate | high | moderate | low | moderate | moderate | moderate |
| Emt31 | low | moderate | low | low | moderate | high | moderate | low | moderate | Moderate/high | moderate |
| Emt 32 | low | moderate | low | low | low | high | moderate | moderate | moderate | moderate | moderate |
| Emt 46 | low | moderate | low | low | moderate | high | moderate | low | moderate | moderate | Moderate-high |
| Emt 53 | low | moderate | low | low | moderate | high | moderate | low | moderate | moderate | moderate |
| Emt55 | low | moderate | low | low | moderate | high | moderate | low | moderate | Moderate-high | Moderate-high |
| Emt57 | low | moderate | low | low | low | moderate | moderate | low | moderate | moderate | Moderate |
| Emt58 | low | moderate | low | low | low | low | moderate | high | moderate | moderate | Moderate/high |
| Emt61 | low | moderate | low | low | low | low | high | moderate | low | moderate | Moderate-high |
